# Landscape‐Wide Metabarcoding Shows Interactions Among the Gut Microbiome and Pollen Diversity in the Invasive Bumblebee, *Bombus terrestris*

**DOI:** 10.1002/ece3.71717

**Published:** 2025-07-07

**Authors:** Sabrina Haque, Hasinika K. A. H. Gamage, Cecilia Kardum Hjort, Fleur Ponton, Francisco Encinas‐Viso, Ian T. Paulsen, Rachael Y. Dudaniec

**Affiliations:** ^1^ School of Natural Sciences Macquarie University Sydney New South Wales Australia; ^2^ ARC Training Centre for Facilitated Advancement of Australia's Bioactives Macquarie University Sydney New South Wales Australia; ^3^ Department of Biology Lund University Lund Sweden; ^4^ Centre for Australian National Biodiversity Research CSIRO Canberra Australian Capital Territory Australia; ^5^ ARC Centre of Excellence in Synthetic Biology Macquarie University Sydney New South Wales Australia

**Keywords:** *Bombus terrestris*, bumblebee, environment, gut microbiome, invasive species, metabarcoding, pollen, Tasmania

## Abstract

Gut microbial communities can facilitate traits that are essential for invasive species survival in novel environments. Despite the global plethora of invasive social insect species, the role of the gut microbiome in colonisation success under novel dietary and environmental conditions is little known. The introduction of the European buff‐tailed bumblebee, *Bombus terrestris* , to the island of Tasmania (Australia) ~30 years ago is of ecological concern due to its negative impacts on native vegetation and endemic bees. Here, we investigate how the gut microbiota of *B. terrestris* workers is affected by corbicular pollen diversity and environmental variation across diverse landscapes in an invaded island system. *B. terrestris* female workers were sampled from 19 sites across Tasmania, for which environmental data for seven variables were extracted. Using 16S rRNA and ITS2 metabarcoding on gut samples and foraged pollen, respectively, we examine how the gut microbiota of *B. terrestris* is influenced by pollen diversity, environmental variables and their interactions. Gut bacterial community composition was significantly predicted by site annual precipitation and the percentage of pasture, which each explained 9% of the variation. Gut bacterial diversity was also explained by precipitation and pasture (40% and 30% of the variation, respectively). Furthermore, a positive interaction between annual precipitation and annual temperature significantly predicted site gut bacterial diversity. The interaction effect of pollen diversity and summer wind velocity was also positively related to gut bacterial diversity. Our findings contribute to understanding how interactions between the local environment and pollen diet affect the bee gut microbiome and thus the health and success of invasive pollinators.

## Graphical abstract

The study examines how environmental factors and dietary diversity influence the gut microbiome of the invasive European buff‐tailed bumblebee ( *Bombus terrestris* ) in Tasmania. We found that gut bacterial composition and diversity were significantly affected by annual precipitation, pasture percentage, temperature and interactions between pollen diversity and wind velocity. These findings highlight how environmental interactions shape the microbiome of invasive bumblebees, providing insights into their health, spread and persistence worldwide.

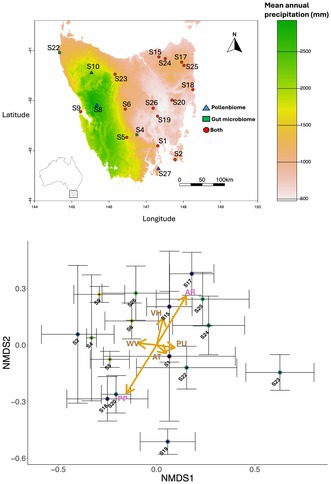

## 1 Introduction

The role of microbial communities in facilitating insect invasions is increasingly recognised, with influences on survival, reproduction and ecological integration in novel environments. However, an understanding of how diet and environmental factors interact to affect host microbiomes and in turn affect fitness and colonisation success is still emerging (Lu et al. 2016; Escalas et al. 2022; Fontaine and Kohl 2020; Zhu et al. 2021). Novel diets and environments can alter gut microbiomes of invasive insects which may influence their health positively by increasing fitness (e.g., Himler et al. 2011; Han et al. 2024) or negatively via reducing gut microbial diversity (e.g., Rosso et al. 2018). Therefore, unravelling the links between environment, nutrition and the gut microbiome of invaders as they establish within novel locations is relevant for understanding how species adapt during biological invasions.

Among terrestrial invaders, insects, especially social and eusocial Hymenoptera (e.g., bees, wasps, ants) are invasive on a global scale (Russo 2016; Manfredini et al. 2019; Ghisbain et al. 2021) with the introductions of pollinators (i.e., bees) into agricultural settings beyond their native ranges being particularly impactful (Russo 2016; Aizen et al. 2020). Introduced bees can compete with native pollinators for floral resources and nesting habitats, can spread pathogens, and they frequently serve as primary pollinators for numerous weeds (Goulson 2003; Hanley and Goulson 2003; Lowenstein et al. 2019; O'Connell et al. 2021). Urban landscapes may offer valuable opportunities for introduced pollinators as they often have high floristic diversity, including many non‐native species (Matteson and Langellotto 2009; Hülsmann et al. 2015; Lowenstein and Minor 2016). The foraging resources available to invasive pollinators may therefore affect both their diet and their microbiomes, with consequences for vegetation communities (Goulson 2003; Hanley and Goulson 2003). Elucidating how environmental factors and novel diets influence the gut microbiome of invasive pollinators is important for understanding how these invaders maintain health and persist within novel ecosystems.

While bee health is tightly linked with the gut microbiome, bee diet (i.e., pollen and nectar) plays a large part (Motta and Moran 2024), and this interaction is likely to be significant when invading diverse novel landscapes (Anderson et al. 2011; Engel et al. 2016). While nectar provides carbohydrates, pollen is the primary source of proteins, lipids, vitamins and minerals essential for bee development, immunity and longevity (Roulston and Buchmann 2000; Brodschneider and Crailsheim 2010; Alaux et al. 2011). Diverse, polyfloral diets offer broader nutritional benefits compared to monofloral diets (Brodschneider et al. 2021) and are critical for functions like royal jelly production (Crailsheim et al. 1992). Pollen can also enhance bees' ability to metabolise toxic compounds, including pesticides (Barascou et al. 2021). Genomic and metagenomic studies show that bee gut bacteria assist in nutrient processing, toxin neutralisation and parasite defence (Engel et al. 2012; Lee et al. 2015; Engel et al. 2016). Thus, examining how pollen‐derived nutrition shapes gut bacterial communities is key to understanding bee health and the factors that may facilitate their invasion success.

In highly social corbiculate (i.e., pollen basket bearing) bee species, the gut microbiome consists of a relatively small and consistent group of coevolved taxa, which play key roles in digestion, growth, immunity and detoxification (Kwong, Medina, et al. 2017). The buff‐tailed bumblebee, *Bombus terrestris* , native to Europe, has become a widespread invasive species in regions such as South America, New Zealand, Japan and Australia (Aizen et al. 2020). *B. terrestris* diverged from honeybees around 80 million years ago and share most core gut bacterial genera with honeybees (Lim et al. 2015; Kwong, Mancenido, and Moran 2017). Bumblebee workers typically inherit microbiota from their founding queen, suggesting vertical transmission (Hammer et al. 2021; Su et al. 2021). The core gut microbiome of *B. terrestris* consists of seven dominant bacterial genera (Hammer et al. 2021), while the environmentally acquired (hereafter ‘facultative’) taxa such as *Apibacter*, *Bartonella*, *Bombella*, *Acetobacter* and *Commensalibacter* are less abundant and are likely acquired through landscape and floral resources (Kwong and Moran 2016; Callegari et al. 2021; Zhang et al. 2023). Understanding the balance between core and facultative taxa is crucial for revealing how gut microbiomes support bumblebee health in novel environments.

The use of DNA metabarcoding to characterise pollen diversity offers a powerful method for investigating the pollen composition of bee diets, and spatial and temporal fluctuations in plant–pollinator interactions (Bell et al. 2022; Milla et al. 2022; Encinas‐Viso et al. 2022). Pollen DNA sequencing and metabarcoding can identify the diversity of plant taxa visited from the pollen grains carried within bee corbicular pollen or attached to the body (Keller et al. 2015; Bell et al. 2017). Metabarcoding studies of pollen have revealed new interactions within flower‐visitor networks, uncovering missing links in the pollination biology of cryptic plant species (Pornon et al. 2017; Lucas et al. 2018; Arstingstall et al. 2021; Encinas‐Viso et al. 2022). Therefore, pollen metabarcoding can enhance our understanding of plant–pollinator interactions and their influence on gut bacterial communities across diverse environments.

Tasmania, the island state of Australia, witnessed a rapid and successful invasion of the European bumblebee, *B. terrestris* , following its introduction in 1992 (Semmens et al. 1993; Hingston 2006a), but it has failed to colonise the Australian mainland. Although the initial colonisation likely involved only a few queens from New Zealand (Schmid‐Hempel et al. 2007), *B. terrestris* spread across the entire island within a decade (Hingston et al. 2002). Despite showing some spatial genetic structuring, the Tasmanian *B. terrestris* population has high gene flow with low genetic diversity (Kardum Hjort et al. 2024). The invasion of *B. terrestris* in Tasmania has raised concerns regarding competition with and displacement of native bees and other pollinators (Hingston and McQuillan 1999; Hingston and Wotherspoon 2017), reduced pollination efficiency of native plants through nectar robbing and physical flower damage (Hingston and McQuillan 1998) and its capacity to spread exotic weeds (Hingston 2006b). Although *B. terrestris* has not yet established on mainland Australia, its high mobility, the availability of suitable habitats, and similar climatic conditions in the southern and eastern coastal regions pose a significant invasion risk (Hingston 2007; Acosta et al. 2016; Kardum Hjort et al. 2024). Overall, the introduction of *B. terrestris* to Tasmania represents an ecological threat due to its capacity to disrupt native plant–pollinator networks and spread invasive weeds.

Here we explore the intricate relationships between the gut microbiome and pollen diversity in the invasive bumblebee, *B. terrestris* , across varying environmental conditions in Tasmania, Australia. Through landscape‐wide sampling and a metabarcoding approach to characterise both gut bacteria (16S: gut microbiome) and diet composition from foraged pollen (ITS2: floral diversity of corbicular pollen), we aim to test the following predictions:

(i) Gut bacterial composition and diversity in *B. terrestris* will vary with environment (i.e., climate, land use), (ii) corbicular pollen diversity will correlate with gut bacterial diversity in *B. terrestris* and show interactions with the environment. By systematically investigating interactions among the gut microbial–dietary interface of an invasive pollinator and its novel environment, we provide landscape‐level insights into what shapes gut microbial communities following host invasion.

## 2 Materials and Methods

### 2.1 Study Design and Bumblebee Collection

**FIGURE 1:**
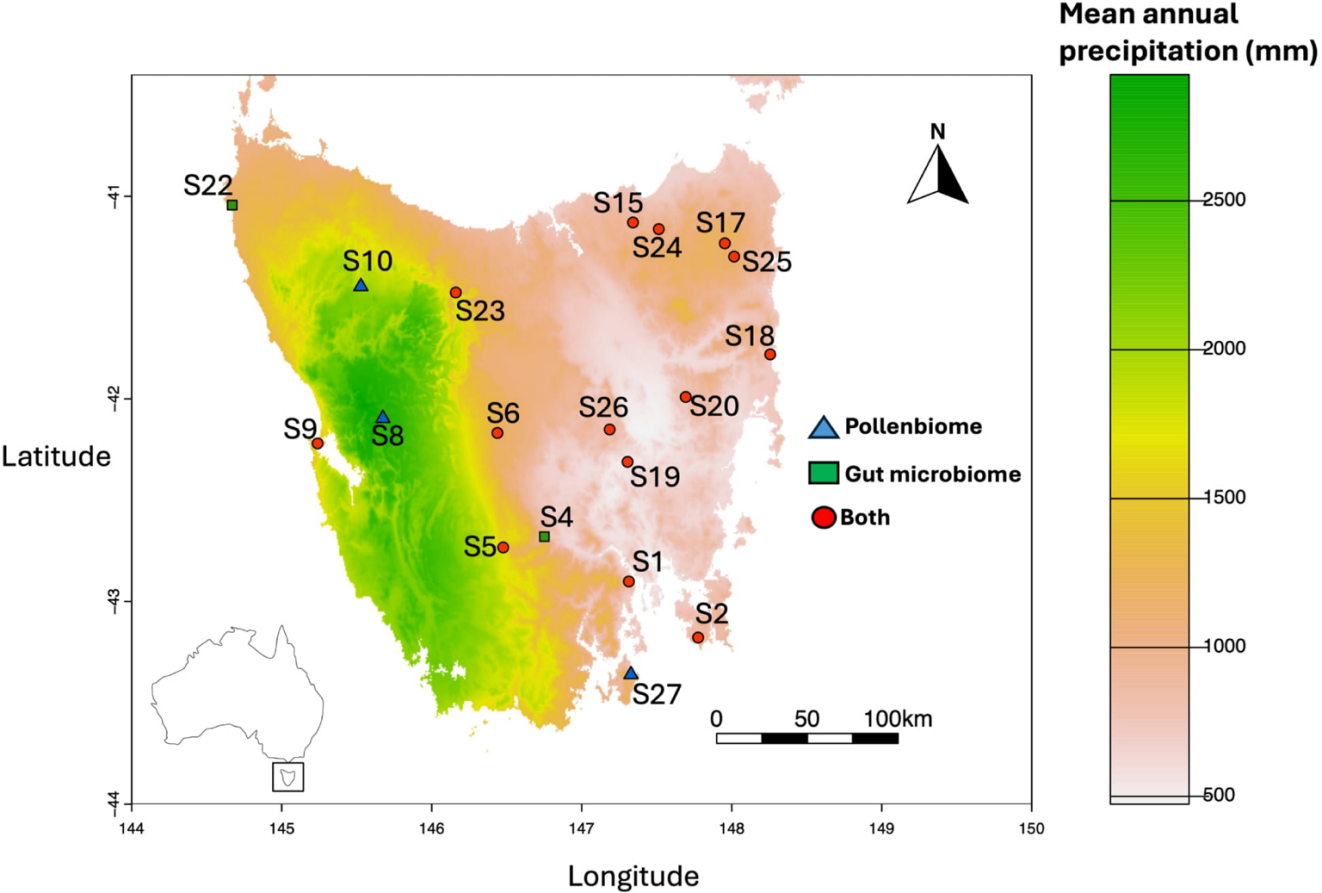
Sampling sites for *B. terrestris* (*n* = 19; see Table 1 for corresponding site names) on a map of mean annual precipitation (mm) across Tasmania, within Australia (inset). Key: ‘Pollen’ = sites exclusively used in the pollen study, ‘Gut’ = sites exclusively involved in the gut microbiome study, ‘Both’ = sites employed in both gut microbiome and pollen studies.

*B. terrestris* female workers were sampled from 19 sites across Tasmania, the Australian island state (Table 1, Figure 1), with site selection informed by prior records of the species occurrence (Hingston et al. 2002; Hingston 2006a, 2006b). Bumblebees were typically collected from open areas of residential urban and rural regions, where flowering plants were present (such as road verges, flowery grass patches, coastal meadows, forest and national park margins, gardens and parks), as described by Kardum Hjort et al. (2023, 2024) and Text S1. We obtained gut microbiome data for 16/19 sites (*n* = 6 to 8 bees per site, Table S1), while pollen samples were collected from 17/19 sites (pollen pooled from bees per site) (Table 1, Figure 1). Three sites (S8, S10 and S27) included in the pollen study were not a part of the gut microbiome study because the bees from these sites had been previously damaged and their bodies were not suitable for gut dissection. Conversely, two sites (S4 and S22) from the gut microbiome study were excluded from the pollen study because the bees from these sites did not carry any pollen (Figure 1).

**TABLE 1:** Environmental metadata for each sampling site. Sampling was conducted at a total of 19 sites across Tasmania, Australia.

Site ID	Site name	*N*	AT	AR	PP	PU	VH	WV
S1	Hobart	6	11.96	670.44	0.00	32.34	98.10	5.79
S2	Port Arthur	5	10.75	903.11	4.88	45.70	0.00	5.83
**S4**	**Mount Field**	**4**	**10.34**	**939.74**	**0.00**	**33.84**	**2.22**	**4.23**
S5	South West	6	9.68	1212.35	6.25	28.91	26.25	4.79
S6	Cradle Mountain	6	9.68	1223.70	0.00	22.41	0.00	3.91
*S8*	*Franklin Gordon*	*0*	*10.68*	*2613.83*	*0.00*	*0.58*	*23.58*	*5.32*
S9	Macquarie Heads	6	11.96	1467.57	0.00	26.32	1.94	6.42
*S10*	*Tikkawoppa*	*0*	*8.91*	*2041.05*	*1.38*	*45.34*	*27.91*	*4.91*
S15	Nabowla	4	12.49	873.19	11.88	19.36	2.07	4.71
S17	Weldborough	5	10.22	1167.57	4.35	32.19	3.00	4.22
S18	Douglas‐Apley	5	12.63	691.63	35.13	17.43	2.50	5.23
S19	Oatlands	7	10.61	507.36	8.90	12.28	2.00	4.00
S20	Campbell Town	6	9.61	759.56	57.63	14.05	2.84	4.45
**S22**	**Arthur River**	**7**	**12.57**	**1167.80**	**0.00**	**37.91**	**7.39**	**6.42**
S23	Cethana	7	9.83	1477.95	2.06	37.52	3.77	4.62
S24	Anabels Cottage	6	12.21	1022.65	0.00	18.07	51.23	4.70
S25	Pyengana	6	11.37	1119.97	0.17	19.76	8.08	4.29
S26	Interlaken	6	7.97	803.54	0.00	27.83	2.46	4.13
*S27*	*BrunyIsland*	*0*	*11.39*	*838.75*	*11.06*	*18.54*	*25.23*	*6.18*

*Note:* Cells in bold represent sites exclusively involved in the gut microbiome study (*n* = 16), while cells in italics show sites exclusively used in the pollen study (*n* = 17). All other sites were used in both the gut microbiome and pollen analyses.

Abbreviations: AR, mean annual precipitation (mm); AT, mean annual temperature (°C); *N*, number of *B. terrestris* used for gut microbiome sequencing per site; PP, percentage of pasture (%); PU, percentage of urbanisation (%); VH, height of vegetation (mm); WV, average velocity of summer wind (m/s).

### 2.2 Selection of Environmental Variables

Our 19 sampling sites spanned diverse climates, spanning the drier eastern to wetter western longitudes. The sites were sampled for environmental variables as described in Kardum Hjort et al. (2023, 2024) and summarised in Table 1. Environmental variables including mean annual temperature (°C), mean annual precipitation (mm), precipitation seasonality (mm) and average summer wind velocity (m/s) were obtained from WorldClim v2.1 (Fick and Hijmans 2017). Land cover details such as the percentage of pasture were sourced from The National Dynamic Land Cover Dataset, while vegetation height (m) came from the ICESat Vegetation Height and Structure data set (Scarth 2013). The percentage of urban area was acquired from the Catchment Scale Land Use of Australia dataset (ABARES 2021). These variables were extracted within a circular area around each site with a 1 km^2^ radius. Environmental variables were converted from cell fraction to the percentage of the total 1 km^2^ area (Kardum Hjort et al. 2023; Kardum Hjort et al. 2024).

### 2.3 Environmental Variables Correlation

A Pearson correlation matrix was generated using R version 4.3.1 (R Core Team 2024) to examine the relationships among all environmental variables across the sampled sites. Of all comparisons, only precipitation seasonality (mm) showed a high positive correlation (*r* ≥ 0.7) with mean annual precipitation (mm) and was excluded from the analysis. The final set of predictor variables consisted of six environmental factors: mean annual temperature (°C), mean annual precipitation (mm), percentage of pasture (%), height of vegetation (mm), percentage of urbanisation (%) and average summer wind velocity (m/s) (Table S1).

### 2.4 Gut Bacterial 16S rRNA Metabarcoding and Taxonomic Identification

Gut dissections of the mid and the hind gut were performed for four to eight *B. terrestris* per site for 16 sites (Table 1), and DNA was extracted using a modification of the DNeasy Blood and Tissue Kit protocol (Qiagen), as described in Text S2. DNA metabarcoding of the *B. terrestris* gut microbiome was performed on the V4 region of the 16S rRNA gene for a total of 92 bumblebees (Text S3). The library preparation and sequencing (Text S3) were carried out at the Ramaciotti Centre for Genomics (University of New South Wales, Sydney, Australia), with a 2 × 250 bp paired‐end run on an Illumina MiSeq platform. Bioinformatics was performed on raw sequence data using Quantitative Insights into Microbial Ecology (QIIME‐2, v2022.8; Bolyen et al. 2019; Estaki et al. 2020). Demultiplexed paired‐end reads were quality filtered using the q2‐demux plugin, followed by denoising with Deblur (Amir et al. 2017). All amplicon sequence variants (ASVs) were aligned with mafft, via q2‐alignment (Katoh et al. 2002) and used to construct a phylogeny with fasttree2, via q2‐phylogeny (Price et al. 2010). The generated ASVs were assigned to taxonomy using a pretrained Naïve Bayes classifier against the Silva‐138 reference database (Quast et al. 2012; Yilmaz et al. 2014; Bolyen et al. 2019) of the 515F/R806 region of the 16S rRNA gene and the q2‐feature‐classifier plugin (Bokulich et al. 2018; Robeson et al. 2021).

### 2.5 16S rRNA Data Filtering and Taxonomic Classification

Sequencing of the bacterial 16S rRNA amplicons yielded 9,679,597 reads (*n* = 92 bumblebees from 16 sites). Following demultiplexing and quality filtering, a total of 6,396,272 reads were retained. Sequences shorter than 160 bp were excluded, and the sequence data were rarefied at a sequencing depth of 40,000 reads per sample, generating a total of 708 ASVs. We retained all 708 ASVs for analyses of community composition (e.g., NMDS and pairwise PERMANOVA) and for all statistical tests (e.g., alpha diversity and LME models). Taxonomic assignment of ASVs was performed using a Naïve‐Bayes classifier trained on the SILVA‐138 reference database. To calculate the relative abundance of core and facultative gut bacterial genera, we filtered out low‐abundance taxa, retaining genera with a relative abundance greater than 1% across all sites. Genera below 1% were excluded because they contribute very little to the overall community structure and would not provide reliable resolution for visualisation in bar plots and heatmaps. Core and facultative assignments were based on these major taxa (> 1% relative abundance).

### 2.6 Statistical Analyses of 16S rRNA Data

To explore spatial variation in gut bacterial composition, Bray–Curtis dissimilarities were analysed and calculated using ASV abundance data. An initial nonmetric multidimensional scaling (NMDS) was performed for all individual *B. terrestris* workers (*n* = 92) using the *vegan* R package version 2.6‐4 (Oksanen et al. 2024). Environmental vectors—mean annual temperature, mean annual precipitation, percentage of pasture, percentage of urbanisation, height of vegetation and average summer wind velocity—were overlaid, and their correlations were calculated to identify significant environmental predictors of community variation. This step was conducted on the full sample‐level dataset to avoid bias related to site‐level averaging and to capture within‐site variability. To visualise broader patterns and reduce visual complexity, a second NMDS was generated using site‐level data (*N* = 16 sites in the gut microbiome study), with ASV abundances averaged across individuals per site, which was plotted with centroids and 95% confidence intervals (standard error bars). The same environmental vectors were re‐overlaid, and correlations were re‐assessed to confirm consistency with the individual‐level NMDS results, enabling clearer depiction of ecological trends at landscape scale.

To statistically test for spatial variation in gut microbial composition across our study area, a pairwise permutational multivariate analysis of variance (PERMANOVA) was conducted to ascertain pairwise site differences using the *vegan* and *pairwiseAdonis* R packages version 0.4.1 (Martinez Arbizu 2020). Pairwise PERMANOVA was performed with *Bonferroni* correction, applied for multiple comparisons. To verify that PERMANOVA assumptions were met, we tested for homogeneity of group dispersions using the *betadisper* and *permutest* functions in *vegan*. No significant differences in dispersion were detected (permutest: *p* > 0.05), confirming that group‐level differences in composition were not driven by unequal within‐group variability. Alpha diversity was calculated using Chao1 richness and Shannon's diversity indices of gut bacteria using the *phyloseq* R package version 1.44.0 (McMurdie and Holmes 2013), which captured species richness and diversity, respectively. As this version of *phyloseq* did not support analysis of variance (ANOVA) test for Chao1 richness, *t*‐tests were conducted to assess differences in species richness across sites, while ANOVA was used for Shannon's diversity. Significant site‐specific differences in gut microbial community composition, diversity and richness were assessed for spatial patterns that might indicate effects of the local environment or other site characteristics on the gut microbiome.

### 2.7 Pollen DNA Extraction and ITS2 Metabarcoding

Pollen was removed from each bee as described in Text S4, with pollen being pooled from all bees collected at a site. The pooled pollen samples from each site were extracted for DNA using a modified protocol with the NucleoSpin Food Kit (Macherey Nagel), as described in Text S4. Quantification of the extracted DNA was conducted using a Qubit 4 Fluorometer with the dsDNA HS Assay Kit (Invitrogen). We used a metabarcoding approach to characterise corbicular pollen diversity using the Internal Transcribed Spacer region 2 (ITS2), which is a variable DNA region in plants (Chen et al. 2010), spanning 100 to 700 bp. This barcode is known for its remarkable discriminatory capabilities in pollen metabarcoding studies, particularly at the genus level (Yao et al. 2010; Milla et al. 2022). Metabarcoding is a highly comparable, time‐efficient approach that is found to be more repeatable and sensitive over traditional microscopy (i.e., melissopalynology), which is heavily reliant on taxonomic expertise (Hawkins et al. 2015). PCR was conducted on the extracted pollen DNA samples to amplify the ITS2 region as described in Text S4. PCR products were subjected to purification, followed by a secondary PCR clean‐up, library preparation and 2 × 250 bp paired‐end sequencing performed on an Illumina MiSeq platform (Text S3).

### 2.8 Data Filtering of Pollen ITS2 Data and Plant Taxa Identification

Pollen samples from 17 sites yielded 1,100,167 forward and reverse reads, which were quality filtered, trimmed, merged and processed using the DADA2 ITS pipeline (Callahan et al. 2017). Primer sequences were identified and removed using *cutadapt* version 0.4 (Martin 2011) prior to proceeding with the DADA2 ITS standard protocol. After chimera removal, 416,268 reads retained, and a table with 865 ASVs were generated. ASVs with fewer than 10 observations were removed from the table, resulting in a final count of 445 ASVs. Plant genera were assigned to ASVs using BLAST (e‐value ≤ 1 × 10^−50^, ≥ 90% identity) with special emphasis on Tasmanian flora, grouped into three categories: (i) ‘native’ (and endemic) Australian plant genera, (ii) ‘introduced’ (or invasive) plant genera in Tasmania, and (iii) a ‘both’ category, which included plant genera containing both native and introduced species in Tasmania (Key to Tasmanian Vascular Plants 2023; Australasian Virtual Herbarium 2023). This classification was used to investigate which type of plant was most foraged by the invasive *B. terrestris* (Table S2). Euclidean distance matrices were used to explore site‐level similarities in foraged plant communities.

### 2.9 Interactions Between Gut Bacteria, Pollen and Environmental Variables

Interactions of Shannon's diversity indices of the gut microbiome and pollen (response variables) with environmental factors (predictor variables) were analysed using the *lme* function in the *nlme* R package (version 3.1–166) (Pinheiro et al. 2024). Linear mixed effect models were used with the *lme* function to investigate the: (1) impact of pollen diversity on gut bacterial diversity, (2) the influence of environmental variables on gut bacterial diversity and (3) the interaction effects of environmental variables and pollen diversity on gut bacterial diversity. The models were then compared based on AIC using the *dredge* function within the *MuMIn* R package (version 1.47.5) (Bartoń 2023). To achieve this, the percentage of pasture values first underwent logit transformation to linearise the relationship before conducting the *dredge* analysis. The sum of weights for the *dredge* models was calculated using the *sw* function in the *MuMIn* R package. Similarly, the interactions between Chao1 richness of gut bacteria, Chao1 richness of pollen, and environmental variables were also analysed using linear mixed effect models. However, comparisons based on AIC and sum of weights were not conducted between Chao1 richness indices of gut bacteria, pollen and environment due to their lack of significance in the *lme* analysis (Table S3).

## 3 Results

### 3.1 Gut Bacterial Taxa Across Sites

**FIGURE 2:**
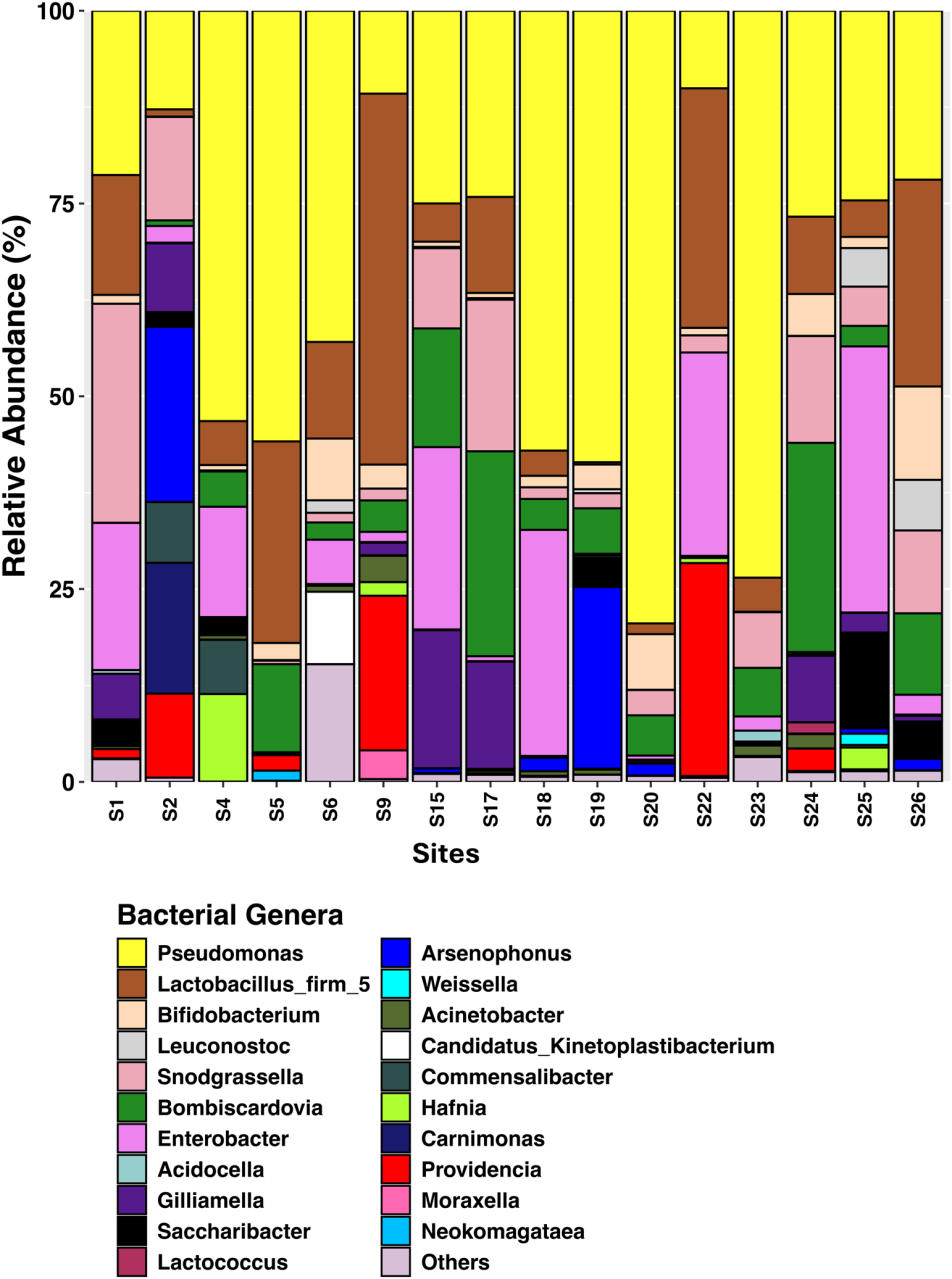
Relative abundance of major gut bacterial genera from *B. terrestris* per site. ‘Others’ indicate all bacterial genera that contributed less than 1%.

**FIGURE 3:**
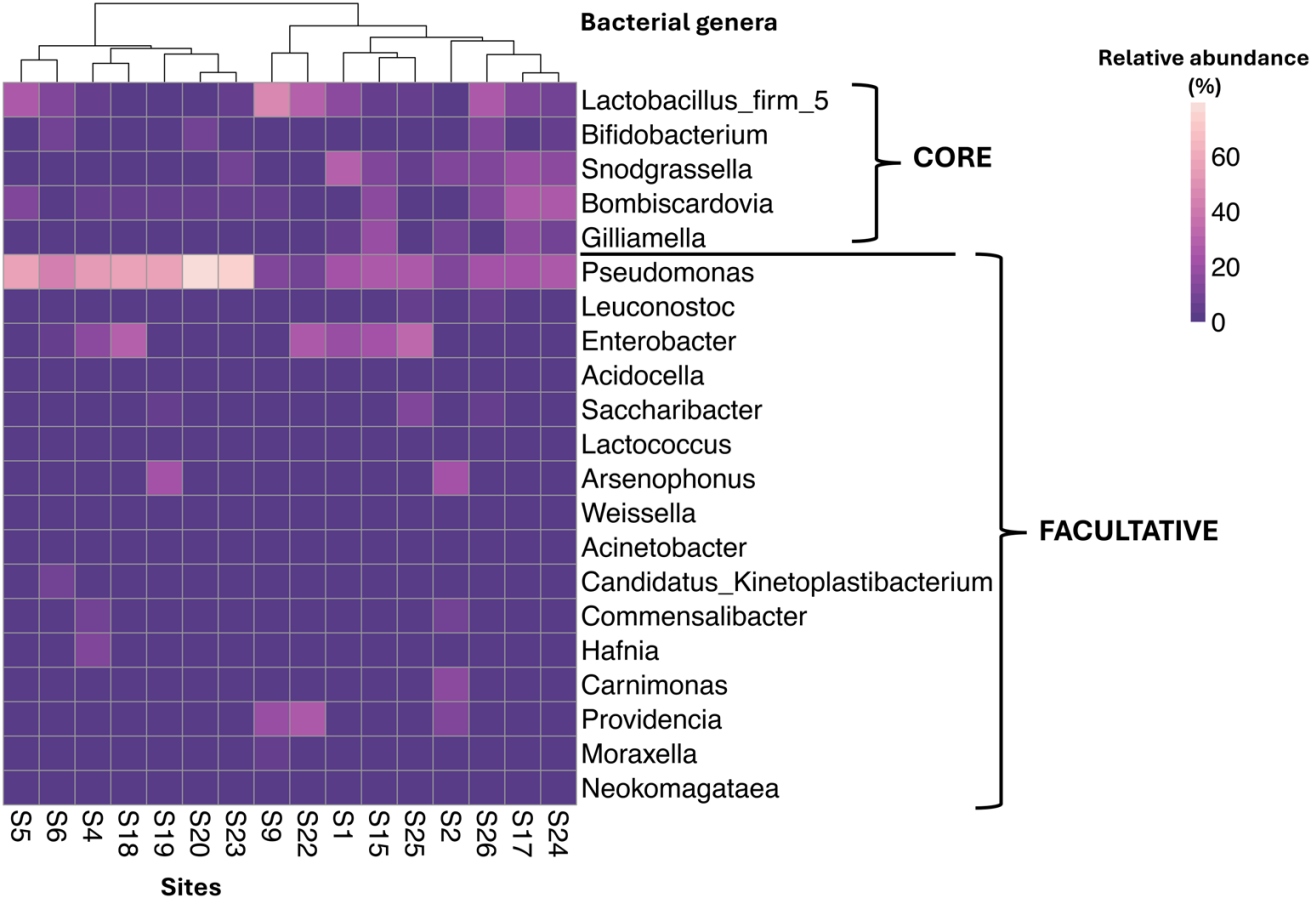
Heatmap showing the relative abundance of core and facultative members of gut bacteria within *B. terrestris* across Tasmania. The colour scale shows the relative abundance of bacterial genera (> 1%) within each site. The dendrogram on top of the heatmap depicts the distance or similarities among sites, which is constructed based on outcomes of the hierarchical clustering calculation using the Euclidean distance matrix, revealing the specific nodes to which each site is assigned.

Proteobacteria, Firmicutes and Actinobacteriota constituted the primary gut bacterial phyla in the bumblebee workers across all 16 sites (Figure S1A). Proteobacteria were the most abundant phyla in most sites, except for S9 (43.9%), where Firmicutes were prevalent (48.7%) (Figure S1A). A total of 16 major gut bacterial families were identified across the sites (Figure S1B). Analysis of relative abundance data (Figure 2) revealed that five core gut bacterial genera—*Lactobacillus Firm‐5*, *Snodgrassella*, *Bombiscardovia*, *Gilliamella* and *Bifidobacterium*—were consistently detected across *B. terrestris* samples (Figure 3) aligning with other bumblebee microbiome studies (e.g., Hammer et al. 2021). All other detected genera were classified as ‘facultative’ taxa.

The heatmap‐dendrogram based on Euclidean distances (Figure 3) clustered the 16 sampling sites into three major groups according to their gut bacterial community profiles. The first group consisted of sites S5, S6 and S4, primarily characterised by elevated relative abundances of the core bacteria *Lactobacillus, Firm‐5* and *Bifidobacterium*. The second group, comprising sites S18, S19, S20, S23, S9 and S22, showed higher relative abundances of the core bacteria *Snodgrassella* and *Gilliamella*. Particularly, sites S9 and S22 were distinguished by higher levels of the core genus *Lactobacillus Firm‐5* (48.1% and 31.1%, respectively; Figures 2 and 3), consistent with their distinct clustering in the dendrogram. The third notable group included sites such as S1, S15, S25, S2, S26, S17 and S24, which exhibited greater relative abundance of core genera, but also representation of facultative genera including *Pseudomonas* and *Enterobacter*. Overall, while core gut taxa dominated the proportion of gut bacteria detected at most sites, variations in the abundance of facultative taxa contributed to the observed spatial structuring of gut bacterial communities.

### 3.2 Gut Bacterial Community Composition and Environmental Variables

**FIGURE 4:**
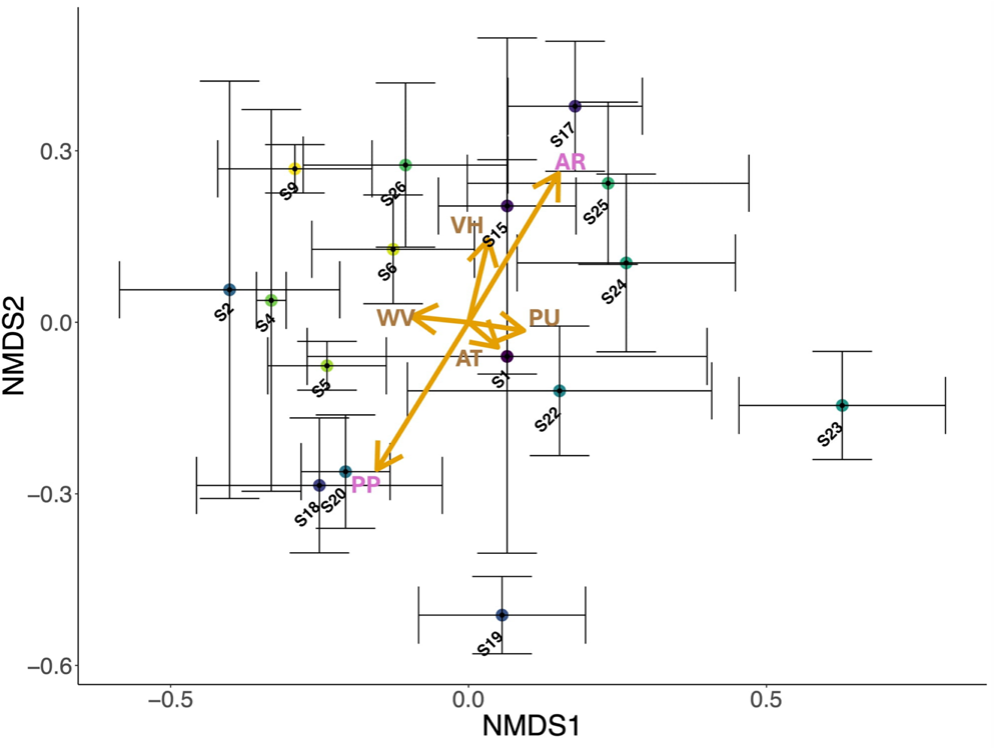
Ordination of gut bacterial communities for *B. terrestris* across 16 sites. Stress = 0.20. NMDS was constructed based on Bray–Curtis dissimilarity matrix of ASV abundance. Circles depict the centroid of ASV communities at each site (mean ASV abundance per site), with 95% confidence intervals. AR, mean annual precipitation (mm); AT, mean annual temperature (°C); PP, percentage of pasture (%); PU, percentage of urbanisation (%); VH, height of vegetation (mm); WV, velocity of summer wind (m/s) (see Table S3 for corresponding environmental vector correlations).

NMDS ordination (Figure 4) revealed that the community composition of *B. terrestris* gut bacteria was significantly associated with the percentage of pasture (*r* ^2^ = 0.090; *p* = 0.02, Table S4) and mean annual precipitation at sites (*r* ^2^ = 0.091; *p* = 0.01, Table S4). These environmental associations were consistent with those observed in the initial NMDS analysis, which was carried out using individual‐level data (Figure S2), indicating that site‐level averaging did not bias the detected patterns in correlation, indicating that pasture and precipitation are the key drivers of spatial variation in *B. terrestris* gut microbiome composition across the Tasmanian landscape.

Pairwise PERMANOVA analysis revealed significant spatial variation in gut bacterial community composition across sites. In *B. terrestris*, 49 out of 120 pairwise site comparisons (40.8%) showed significant differences (*p* ≤ 0.05, Table S5), with sites S9 and S22 standing out as the most distinct—differing significantly in 13/15 and 12/15 comparisons, respectively (Table S5). However, S9 and S22 were not significantly different from each other (*p* = 0.194, Table S5) and both were characterised by high relative abundances of *Lactobacillus* Firm‐5 (S9 = 48.1%, S22 = 31.1%, Figures 2 and 3). These two sites were also geographically isolated along Tasmania's western coast. Significant site‐level differences suggest that local environmental conditions, landscape variation, or differences in foraged floral resources may drive spatial structuring of the gut microbiome.

### 3.3 Alpha Diversity of Gut Bacteria Across Sites

Diversity analyses indicated that sites S23 and S25 had the highest alpha diversity of *B. terrestris* gut bacteria (Figure S3). However, pairwise *t*‐tests for Chao1 richness revealed no significant differences across sites (all *p* > 0.05, Table S6), indicating consistency in bacterial richness across the landscape. In contrast, Shannon's diversity differed significantly in only 2 out of 120 pairwise site comparisons (1.67%), with site T9 showing significant variations from sites S19 (ANOVA: *p* = 0.010, Table S7) and S20 (ANOVA: *p* = 0.032, Table S7).

### 3.4 Plant Taxonomic Composition

**FIGURE 5:**
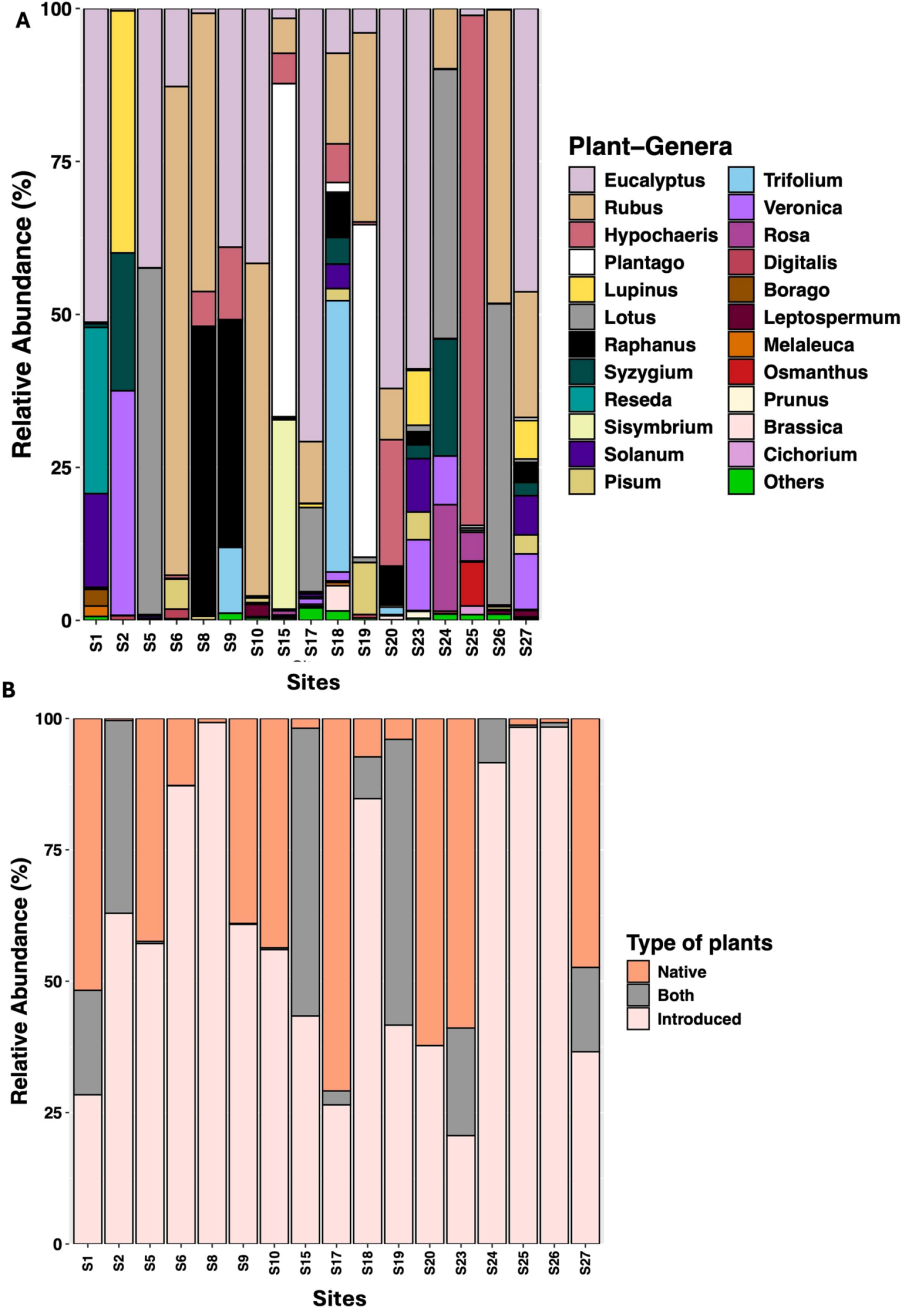
(A) Relative abundance of major plant genera identified from *B. terrestris* corbicular pollen across each sampling site. ‘Others’ indicate the relative abundance of all plant genera that contribute less than 1%. (B) Distribution of type of plants foraged by *B. terrestris* across each Tasmanian site. Explanation of type of plant category: ‘Native’ = plant genera that are native to Tasmania, ‘Introduced’ = plant genera that are exotic and have been naturalised in Tasmania, ‘Both’ = plant genera consisting of both native and introduced species in Tasmania (see Table S6 for plant genera within each category).

A total of 51 plant genera were detected from *B. terrestris* corbicular pollen across 17 sites (Table S2, Figure S4). Of these, 23 genera accounted for up to 99% of the total floral abundance (Figure 5A). Heatmap‐dendrogram analysis revealed three major site clusters based on ASV counts (Figure S4). The first cluster (sites S17 and S18) showed high ASV counts for *Rubus* spp., while the second cluster (sites S1, S15, S19, S23, S26 and S27) was dominated by *Eucalyptus* spp. ASVs. Except for S23, these sites were in the dry eastern part of Tasmania. The third cluster included sites with a higher relative abundance of exotic plant genera or genera that have a combination of native and introduced species and showed no clear geographic pattern. Of the 51 genera identified, 35 were introduced, six were native, and 10 included both native and introduced species (Table S2, Figure 5B).

### 3.5 Effects of Pollen Diversity and Environment on Gut Bacterial Diversity

**FIGURE 6:**
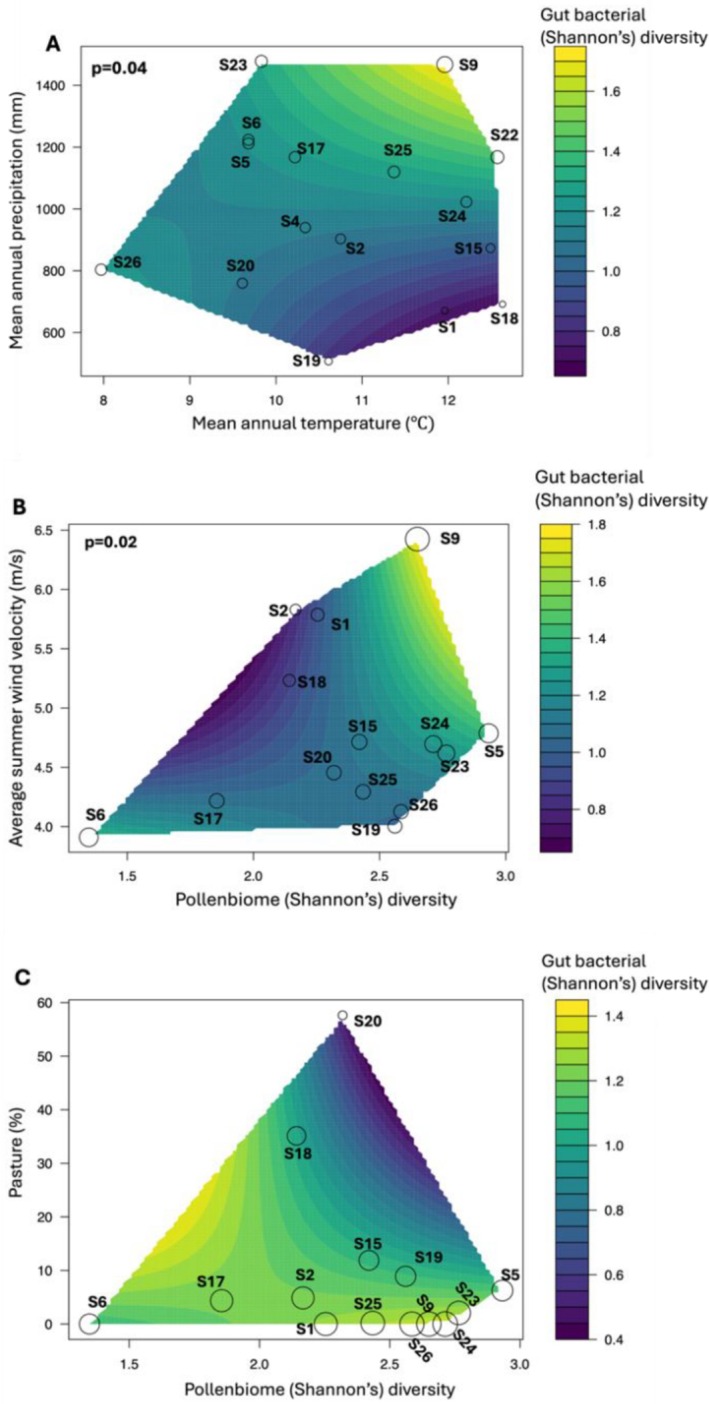
Linear mixed effect models showing (A) a significant positive interaction effect of mean annual precipitation × mean annual temperature on gut bacterial diversity; (B) a significant positive interaction effect of pollen diversity × average summer wind velocity on gut bacterial diversity; and (C) the relationship between percentage of pasture (most strongly weighted variable) and pollen diversity on gut bacterial diversity. In all three plots, the actual paired combinations of x‐axis and y‐axis used to fit the model are depicted as points with circle radii (sites) proportional to average gut bacterial (Shannon's) diversity of *B. terrestris* , and the change in colour scale represents the change in gut bacterial diversity.

Linear mixed‐effect modelling showed that mean annual precipitation and temperature had a significant positive interaction on *B. terrestris* gut bacterial (Shannon's) diversity (*lme*, *p* = 0.04, Table 2, Figure 6A). Gut bacterial diversity also correlated positively with precipitation alone (*lm*, *p* = 0.007, *r* ^2^ = 0.4, Figure S5). Additionally, there was a significant positive interaction between average summer wind velocity and pollen diversity on gut bacterial diversity (*lme*, *p* = 0.02, Table 2, Figure 6B), although no significant linear correlations were found between gut bacterial diversity and pollen diversity or wind velocity when considered separately (*lm*, *p* > 0.05, Figures S6 and S7D).

**TABLE 2:** Linear mixed‐effect models with interactions between *B. terrestris* gut bacterial (Shannon's) diversity, pollen (Shannon's) diversity and environmental variables.

Fixed effect(s)	df	*t* value	*p* value
Bacterial_diversity~AT	12	−0.47	0.65
**Bacterial_diversity~AR**	**12**	**2.87**	**0.01**
**Bacterial_diversity~PP**	**12**	**−2.44**	**0.03**
Bacterial_diversity~PU	12	0.16	0.80
Bacterial_diversity~VH	12	0.58	0.57
Bacterial_diversity~WV	12	0.33	0.75
Bacterial_diversity~AT*AR	**10**	**2.36**	**0.04**
Bacterial_diversity~AT*PP	10	−0.43	0.67
Bacterial_diversity~AT*PU	10	0.28	0.78
Bacterial_diversity~AT*VH	10	−0.75	0.47
Bacterial_diversity~AT*WV	10	1.47	0.17
Bacterial_diversity~AR*PP	10	−0.58	0.58
Bacterial_diversity~AR*PU	10	0.39	0.70
Bacterial_diversity~AR*VH	10	−1.90	0.08
Bacterial_diversity~AR*WV	10	0.99	0.35
Bacterial_diversity~PP*PU	10	0.26	0.80
Bacterial_diversity~PP*VH	10	−1.01	0.33
Bacterial_diversity~PP*WV	10	−0.46	0.65
Bacterial_diversity~PU*VH	10	−1.27	0.23
Bacterial_diversity~PU*WV	10	−1.16	0.27
Bacterial_diversity~VH*WV	10	−2.09	0.06
Bacterial_diversity~Pollen_diversity	12	0.51	0.62
Bacterial_diversity~Pollen_diversity*AT	10	1.67	0.13
Bacterial_diversity~Pollen_diversity*AR	10	−1.52	0.16
Bacterial_diversity~Pollen_diversity*PP	10	−0.76	0.46
Bacterial_diversity~Pollen_diversity*PU	10	0.84	0.42
Bacterial_diversity~Pollen_diversity*VH	10	0.32	0.76
Bacterial_diversity~Pollen_diversity*WV	**10**	**2.76**	**0.02**

*Note:* Cells d in bold show statistically significant interactions with *p* ≤ 0.05. The asterisk (*) denotes a model with main effect and interaction. Random effect ~1|Sites.

Abbreviations: AR, mean annual precipitation (mm); AT, mean annual temperature (°C); DF, degrees of freedom; PP, percentage of pasture (%); PU, percentage of urbanisation (%); VH, height of vegetation (mm); WV, average velocity of summer wind (m/s).

Model selection using Akaike information criterion (AIC) indicated that the top model (ΔAIC = 0, AIC = 131.6, weight = 0.19) included percentage of pasture, pollen diversity and wind velocity, as well as their interaction terms. Percentage of pasture was the strongest predictor of gut bacterial diversity (AIC weight = 1.00), followed by pollen diversity (0.82), pasture × pollen diversity (0.77), wind velocity (0.64) and pollen diversity × wind velocity (0.64; Table 3). Precipitation had a lower weighting (0.53). Pasture percentage had a significant negative correlation on gut bacterial diversity (*lm, p* = 0.03, *r* ^2^ = 0.30, Figure S8). Furthermore, although higher pasture percentage correlated with higher pollen diversity, gut bacterial diversity decreased, driven mainly by two high‐pasture sites, S18 and S20 (Figure 6C).

**TABLE 3:** Comparison of AIC sum of weights for the effect of pollen diversity (Shannon's) and environmental variables on *B. terrestris* gut bacterial diversity (Shannon's).

Variable	Sum of weights	Number of containing models
**logit_pasture**	**1**	**9**
pollen_diversity	0.82	7
logit_pasture: pollen_diversity	0.77	6
wind	0.64	4
pollen_diversity: wind	0.64	4
precipitation	0.53	7
logit_pasture: wind	0.12	1
precipitation: logit_pasture	0.12	2
precipitation: pollen_diversity	0.08	1

*Note:* The most strongly weighted variable is highlighted in bold.

Abbreviations: Logit_pasture, logit transformed values for percentage of pasture; pollen_diversity, Shannon's diversity for pollen samples; precipitation, mean annual precipitation (mm); wind, average summer wind velocity (m/s).

## 4 Discussion

Environmental conditions and floral resources are key drivers of gut microbiome diversity in pollinators, yet their combined effects in invasive species remain poorly understood. Here, we found that for the invasive bumblebee, *B. terrestris, in* Tasmania, increased corbicular pollen diversity at sites with higher percentages of pasture was associated with reduced gut bacterial diversity. Mean annual precipitation showed a positive correlation with the diversity of gut bacteria in *B. terrestris* , whether considered alone or in combination with increasing temperature. Sites characterised by higher average summer wind velocities and greater pollen diversity also exhibited increased gut bacterial diversity in *B. terrestris* . As expected, pollen samples were dominated by introduced plant genera. Moreover, both pasture cover and mean annual precipitation were significant predictors of gut bacterial community composition. These results highlight how environmental factors, and dietary resources interact to shape the gut microbiome of an invasive pollinator, providing insights into the biotic and abiotic processes that influence the health and persistence of invasive bees within novel landscapes.

### 4.1 Environmental Effects on *B. terrestris* Gut Bacteria

We found significant variation in *B. terrestris* gut bacterial composition across sites, with S9 and S22 differing most from others but not from each other (Table S5). This may be related to their geographic isolation on Tasmania's west coast and previously reported genetic differentiation (Kardum Hjort et al. 2024). The percentage of pasture was negatively correlated with gut bacterial community composition (Figure 4) and gut bacterial diversity in *B. terrestris* across Tasmania (Figure S8), which was driven by sites with the highest pasture percentages (i.e., S18 and S20) where intensive agricultural practices likely reduce floristic diversity. Consistent with this, Donkersley et al. (2018) showed that improved grasslands lower gut microbiome diversity in honeybees. Similarly, *B. terrestris* in high‐pasture areas in Tasmania have shorter proboscis lengths, suggesting selection pressures linked to local floral traits (Kardum Hjort et al. 2023). This highlights how agricultural land use can exert ecological pressures on invasive pollinators through alterations in floral resource availability with effects on the gut microbiome.

Mean annual precipitation significantly influenced the gut bacterial community composition of *B. terrestris* (Figure 4). Increased precipitation was correlated with higher gut bacterial diversity (Figure S5), and sites with both high precipitation and temperature also showed enhanced diversity (Figure 6A). Precipitation and humidity are known to impact insect distributions and water regulation (Singh et al. 2009; Koot et al. 2022), and bees are particularly sensitive to drier conditions (Jackson et al. 2018). Seasonal studies on honeybees show that rainy conditions reduce flight activity and alter foraging behaviour (Castelli et al. 2022), likely due to impaired navigation from reduced visual cues (Lawson and Rands 2019). Consequently, changes in foraging patterns under high precipitation may indirectly shape gut microbiomes through altered diets.

Foraged pollen harbours diverse microbial communities that can shift with temperature and humidity, potentially reducing pollen viability and altering bee gut microbiomes (McFrederick and Rehan 2022; Iovane et al. 2022). As pollen microbes are transmitted during foraging, shared pollen and gut microbiomes may enhance bee nutrition and detoxification (Keller et al. 2021). In Tasmania, *B. terrestris* shows local adaptation to precipitation, with candidate genes linked to water loss prevention and olfactory functions (Kardum Hjort et al. 2024), with similar patterns found for *Bombus vancouverensis* (Jackson et al. 2020). These findings suggest that precipitation not only shapes physiological and behavioural traits but may also influence bee–microbiome interactions.

We found that the positive interaction between average summer wind velocity and pollen diversity was significantly associated with increased gut bacterial diversity in *B. terrestris* (Figure 6B), with site S9 exhibiting the highest diversity and strongest winds (Table 1). However, the lack of significant linear correlations between gut bacterial diversity, pollen diversity and wind velocity individually (Figures S6 and S7D) suggests additional factors may be influencing this pattern. The remote coastal location of S9, near the Southern Ocean and Macquarie Harbour, may contribute to these effects. Wind is known to impact bumblebee flight performance (Lawson and Rands 2019; Chang et al. 2016), stability (Ravi et al. 2016), foraging energy expenditure (Crall et al. 2017), guidance to floral rewards (Kantsa et al. 2019) and foraging choices under windy conditions (Mountcastle et al. 2015). Selection signatures related to wind velocity have also been detected in *B. terrestris* populations in Tasmania within a companion study (Kardum Hjort et al. 2024). Together, these findings suggest that environmental factors such as pasture, precipitation and wind not only shape gut bacterial composition but may also drive local adaptation in invasive bee populations.

### 4.2 Diversity and Functions of Core and Facultative Gut Bacterial Taxa

Our study confirmed the presence of the core gut bacterial genera *Snodgrassella*, *Gilliamella*, *Lactobacillus* Firm‐5, *Bombiscardovia* and *Bifidobacterium* in *B. terrestris* (Figures 2 and 3), consistent with previous findings (for e.g., Hammer et al. 2021). These core bacteria can play critical roles in digestion and pathogen defence (Cariveau et al. 2014). Studies have shown that colonisation by *Gilliamella* and *Snodgrassella* can reduce gut infections by *Crithidia bombi*, a trypanosome parasite linked to bumblebee declines (Brown et al. 2003; Otterstatter and Thomson 2008; Engel et al. 2012; Martinson et al. 2011). The abundance of *Lactobacillus* spp. may also increase in *B. terrestris* when infected by *C. bombi* (Blasco‐Lavilla et al. 2024). Biofilm formation on the hindgut by these bacteria may impede pathogen invasion. Core symbionts such as *Bombiscardovia* appear unique to bumblebees, whereas *Snodgrassella* and *Gilliamella* are shared with other social bees (Kwong, Medina, et al. 2017). These microbes contribute to breaking down complex pollen walls and nectar‐derived sugars through enzymes like pectin lyases and glycoside hydrolases (Engel et al. 2012; Zheng et al. 2019; Kešnerová et al. 2020).

Facultative taxa tend to increase with environmental exposure and often replace core microbes under stress (such as pesticides, Kakumanu et al. 2016; Hammer et al. 2021). In addition to core taxa, low levels of noncore (facultative) bacteria, including *Pseudomonas, Commensalibacter*, *Apibacter* and *Arsenophonus*, were detected (Figures 2 and 3). *Pseudomonas*, in particular, was among the major genera detected in our study, mirroring findings in *Bombus bellicosus* where *Pseudomonas* abundance was linked to environmental stressors (e.g., pesticides) and potential dysbiosis (Hammer et al. 2021; De Landa et al. 2023). In our samples, unmeasured stressors may have contributed to the high proportion of *Pseudomonas*, but this would require further study. Other opportunistic taxa like *Hafnia* sp. can dominate in bees with depleted core symbionts, sometimes acting as pathogens (Hammer et al. 2021). Thus, maintaining a healthy core gut microbiome is crucial for digestion, immunity and overall bee health.

### 4.3 Introduced Bee Response to Novel Floral Resources

Two important aspects that may assist bees to persist successfully as invaders are adequate nutrition (via floristic resources) and a functional worker gut microbiota (Kwong and Moran 2016; Dolezal and Toth 2018; Bonilla‐Rosso and Engel 2018). Foraging bees rely on diverse pollen resources for essential proteins, lipids, vitamins and minerals (Branchiccela et al. 2019; Yokota et al. 2024) that depend on the gut microbiome for nutritional uptake and are thus vital for overall growth and development (Brodschneider and Crailsheim 2010; Wright et al. 2018). Notably, the availability of essential amino acids in pollen may affect honeybee foraging preferences (Cook et al. 2003). Digestion of pollen is complex (Wright et al. 2018), and the gut bacteria of bees play a critical role in breaking down complex cell wall polysaccharides such as hemicellulose and pectin, which is essential for accessing the nutrients within pollen (Lee et al. 2015; Lee et al. 2018; Zheng et al. 2019). The interaction is also dynamic; pollen diets with high fatty acid content may help to inhibit the growth of infectious microorganisms (e.g., *American foulbrood* in honeybees, Manning 2001), but alterations to the gut microbiota from malnutrition or disease can reduce the efficiency of protein digestion in honeybees (du Rand et al. 2020).

Nutritional stress due to habitat loss is a key threat to bees (Naug 2009; Goulson et al. 2015) and so may also impact the success of invasive bees in competitive, novel environments (Page and Williams 2023). Since introduced bees typically prefer foraging on introduced plants, they often act as the primary pollinators for several weeds (Lowenstein et al. 2019; O'Connell et al. 2021). Our study revealed that out of 51 plant genera identified from corbicular pollen from *B. terrestris* (Figure S4), the majority (*n* = 35 genera) were introduced (dominated by *Rubus*, blackberry), 10 were native (dominated by *Eucalyptus*), and six genera that occurred as native or introduced species in Tasmania (Table S2, Figure 5B). In Tasmania, several exotic plants such as *Lupinus, Trifolium* and *Raphanus* remained scarce for many decades until the introduction of *B. terrestris* (Hanley and Goulson 2003). Here, we detected several invasive weeds in corbicular pollen including *Rubus, Hypochaeris, Lupinus, Trifolium, Sisymbrium, Digitalis* and *Cirsium* (Figures 5A and S4). The distribution and abundance of some introduced bees are closely tied to the availability of alien food plants. For instance, *Bombus ruderatus* and *Bombus subterraneus* in New Zealand experienced significant declines following changes in agricultural practices that reduced the distribution of invasive *Trifolium pratense* and *Lotus corniculatus* (Macfarlane and Gurr 1995; Hanley and Goulson 2003). However, the generalist *B. terrestris* exhibits a much more generalist foraging habit compared to many other introduced bee species, allowing them to thrive on a suite of non‐native and native plants in Tasmania (Hingston et al. 2002; Goulson 2003).

## 5 Conclusion

Our study marks the first landscape‐scale investigation of the interplay between the gut microbiome and pollen diet in an unmanaged bee pollinator following a recent (~30 years) invasion. Our findings set a foundation for integrating biotic and abiotic processes into the health assessment of invasive or managed pollinators, as well as those of conservation concern. We acknowledge that the data represent a snapshot in time and therefore could be improved with temporal sampling of both pollen and gut microbiomes throughout the foraging period. Notably, how diversity and composition of the bee gut microbiome affect bee health and fitness remains largely unresolved; therefore, our study sets the stage for future experimental approaches to untangle mechanistic effects. Furthermore, our study provides a basis for comparative, interspecific analyses of invasive bumblebee gut microbiomes with that of honeybees and native Australian bees. Here, we establish how environmental interactions influence the gut microbiome of a pollinator across a diverse landscape with varying nutritional resources, which may help to predict the spread and success of invasive pollinators.

## Author Contributions

**Sabrina Haque:** data curation (lead), formal analysis (lead), investigation (equal), methodology (equal), validation (equal), visualization (equal), writing – original draft (lead), writing – review and editing (equal). **Hasinika K. A. H. Gamage:** formal analysis (supporting), methodology (supporting), resources (supporting), supervision (supporting), writing – review and editing (supporting). **Cecilia Kardum Hjort:** data curation (supporting), methodology (equal), resources (supporting), writing – review and editing (supporting). **Fleur Ponton:** investigation (supporting), methodology (supporting), project administration (supporting), supervision (supporting), writing – review and editing (supporting). **Francisco Encinas‐Viso:** funding acquisition (supporting), methodology (supporting), writing – review and editing (supporting). **Ian T. Paulsen:** project administration (supporting), resources (supporting), writing – review and editing (supporting). **Rachael Y. Dudaniec:** conceptualization (lead), data curation (equal), formal analysis (supporting), funding acquisition (lead), investigation (lead), methodology (equal), project administration (lead), resources (lead), supervision (lead), writing – original draft (supporting), writing – review and editing (equal).

## Conflicts of Interest

The authors declare no conflicts of interest.

## Supporting information


Appendix S1.

## Data Availability

Genetic data sets for 16S rRNA (PRJNA1163040) and ITS2 (PRJNA1163066) are submitted to the NCBI BioProject database. Tables of ASVs for 16S and ITS2 data and alpha diversity values for each sample are available on DRYAD at this link: https://doi.org/10.5061/dryad.s7h44j1gw.

## References

[ece371717-bib-0001] ABARES . 2021. Catchment Scale Land Use of Australia – Commodities – Update December 2020. Australian Bureau of Agricultural and Resource Economics and Sciences. 10.25814/jhjb-c072.

[ece371717-bib-0002] Acosta, A. L. , T. C. Giannini , V. L. Imperatriz‐Fonseca , and A. M. Saraiva . 2016. “Worldwide Alien Invasion: A Methodological Approach to Forecast the Potential Spread of a Highly Invasive Pollinator.” Public Library of Science 11, no. 2: e0148295. 10.1371/journal.pone.0148295. PMC4755775 26882479

[ece371717-bib-0003] Aizen, M. A. , M. P. Arbetman , N. P. Chacoff , et al. 2020. “Invasive Bees and Their Impact on Agriculture.” Advances in Ecological Research 63: 49–92. 10.1016/bs.aecr.2020.08.001.

[ece371717-bib-0004] Alaux, C. , C. Dantec , H. Parrinello , and Y. Le Conte . 2011. “Nutrigenomics in Honeybee: Digital Gene Expression Analysis of Pollen's Nutritive Effects on Healthy and *Varroa*‐Parasitized Bees.” BMC Genomics 12: 496. 10.1186/1471-2164-12-496. 21985689 PMC3209670

[ece371717-bib-0005] Amir, A. , D. McDonald , J. A. Navas‐Molina , et al. 2017. “Deblur Rapidly Resolves Single‐Nucleotide Community Sequence Patterns.” MSystems 2, no. 2: e00191–16. 10.1128/mSystems.00191-16. 28289731 PMC5340863

[ece371717-bib-0007] Anderson, K. E. , T. H. Sheehan , B. J. Eckholm , B. M. Mott , and G. DeGrandi‐Hoffman . 2011. “An Emerging Paradigm of Colony Health: Microbial Balance of the Honey Bee and Hive ( *Apis mellifera* ).” Insectes Sociaux 58, no. 4: 431–444. 10.1007/s00040-011-0194-6.

[ece371717-bib-0008] Arstingstall, K. A. , S. DeBano , X. Li , et al. 2021. “Capabilities and Limitations of Using DNA Metabarcoding to Study Plant‐Pollinator Interactions.” Molecular Ecology 30: 5266–5297. 10.1111/mec.16112. 34390062

[ece371717-bib-0009] Australasian Virtual Herbarium – AVH . 2023. “Council of Heads of Australasian Herbaria. Website.” https://avh.chah.org.au.

[ece371717-bib-0010] Barascou, L. , D. Sene , A. Barraud , et al. 2021. “Pollen Nutrition Fosters Honeybee Tolerance to Pesticides.” Royal Society Open Science 8: 210818. 10.1098/rsos.210818. 34540259 PMC8437229

[ece371717-bib-0011] Bartoń, K. 2023. “MuMIn: Multi‐Model Inference.” R Package Version 1.47.5. 10.32614/CRAN.package.MuMIn.

[ece371717-bib-0012] Bell, K. L. , J. Fowler , K. S. Burgess , et al. 2017. “Applying Pollen DNA Metabarcoding to the Study of Plant‐Pollinator Interactions.” Applications in Plant Sciences 5, no. 6: 1600124. 10.3732/apps.1600124. PMC5499302 28690929

[ece371717-bib-0013] Bell, K. L. , K. J. Turo , A. Lowe , et al. 2022. “Plants, Pollinators, and Their Interactions Under Global Ecological Change: The Role of Pollen DNA Metabarcoding.” Molecular Ecology 32, no. 23: 6345–6362. 10.1111/mec.16689. 36086900 PMC10947134

[ece371717-bib-0015] Blasco‐Lavilla, N. , A. López‐López , P. De la Rúa , and S. M. Barribeau . 2024. “Infection by *Crithidia bombi* Increases Relative Abundance of *Lactobacillus* spp. in the Gut of *Bombus Terrestris* .” Molecular Ecology 33, no. 17: e17478. 10.1111/mec.17478. 39075965

[ece371717-bib-0017] Bokulich, N. A. , B. D. Kaehler , J. R. Rideout , et al. 2018. “Optimizing Taxonomic Classification of Marker‐Gene Amplicon Sequences With QIIME 2's q2‐Feature‐Classifier Plugin.” Microbiome 6: 90. 10.1186/s40168-018-0470-z. 29773078 PMC5956843

[ece371717-bib-0018] Bolyen, E. , J. R. Rideout , M. R. Dillon , et al. 2019. “Reproducible, Interactive, Scalable and Extensible Microbiome Data Science Using QIIME 2.” Nature Biotechnology 37: 852–857. 10.1038/s41587-019-0209-9. PMC7015180 31341288

[ece371717-bib-0019] Bonilla‐Rosso, G. , and P. Engel . 2018. “Functional Roles and Metabolic Niches in the Honey Bee Gut Microbiota.” Current Opinion in Microbiology 43: 69–76. 10.1016/j.mib.2017.12.009. 29309997

[ece371717-bib-0020] Branchiccela, B. , L. Castelli , M. Corona , et al. 2019. “Impact of Nutritional Stress on the Honeybee Colony Health.” Scientific Reports 9, no. 1: 10156. 10.1038/s41598-019-46453-9. 31300738 PMC6626013

[ece371717-bib-0021] Brodschneider, R. , and K. Crailsheim . 2010. “Nutrition and Health in Honeybees.” Apidologie 41: 278–294. 10.1051/apido/2010012.

[ece371717-bib-0022] Brodschneider, R. , E. Kalcher‐Sommersguter , S. Kuchling , et al. 2021. “CSI Pollen: Diversity of Honeybee Collected Pollen Studied by Citizen Scientists.” Insects 12, no. 11: 987. 10.3390/insects12110987. 34821788 PMC8625907

[ece371717-bib-0023] Brown, M. J. F. , R. Schmid‐Hempel , and P. Schmid‐Hempel . 2003. “Strong Context‐Dependent Virulence in a Host‐Parasite System: Reconciling Genetic Evidence With Theory.” Journal of Animal Ecology 72: 994–1002. 10.1046/j.1365-2656.2003.00770.x.

[ece371717-bib-0024] Callahan, B. , P. McMurdie , M. Rosen , et al. 2017. “DADA2: High‐Resolution Sample Inference From Illumina Amplicon Data.” Nature Methods 13: 581–583. 10.1038/nmeth.3869. PMC4927377 27214047

[ece371717-bib-0025] Callegari, M. , E. Crotti , M. Fusi , et al. 2021. “Compartmentalization of Bacterial and Fungal Microbiomes in the Gut of Adult Honeybees.” npj Biofilms and Microbiomes 7, no. 42: 1–15. 10.1038/s41522-021000212-9. 33963194 PMC8105395

[ece371717-bib-0026] Cariveau, D. P. , J. E. Powell , H. Koch , J. Elijah Powell , R. Winfree , and N. A. Moran . 2014. “Variation in Gut Microbial Communities and Its Association With Pathogen Infection in Wild Bumble Bees (*Bombus*).” ISME Journal 8, no. 12: 2369–2379. 10.1038/ismej.2014.68. 24763369 PMC4260702

[ece371717-bib-0027] Castelli, L. , B. Branchiccela , H. Romero , P. Zunino , and K. Antúnez . 2022. “Seasonal Dynamics of the Honey Bee but Microbiota in Colonies Under Subtropical Climate: Seasonal Dynamics of Honey Bee Gut Microbiota.” Microbial Ecology 83, no. 2: 492–500. 10.1007/s00248-021-01756-1. 33973059

[ece371717-bib-0028] Chang, J. J. , J. D. Crall , and S. A. Combes . 2016. “Wind Alters Landing Dynamics in Bumblebees.” Journal of Experimental Biology 219, no. 18: 2819–2822. 10.1242/jeb.137976. 27436135

[ece371717-bib-0030] Chen, S. , H. Yao , J. Han , et al. 2010. “Validation of the ITS2 Region as a Novel DNA Barcode for Identifying Medicinal Plant Species.” Public Library of Science 5, no. 1: e8613. 10.1371/journal.pone.0008613. PMC2799520 20062805

[ece371717-bib-0031] Cook, S. M. , C. S. Awmack , D. A. Murray , and I. H. Williams . 2003. “Are Honeybees' Foraging Preferences Affected by Pollen Amino Acid Composition?” Ecological Entomology 28, no. 5: 622–627. 10.1046/j.1365-2311.2003.00548.x.

[ece371717-bib-0033] Crailsheim, K. , L. H. W. Schneider , N. Hrassnigg , et al. 1992. “Pollen Consumption and Utilization in Worker Honeybees ( *Apis mellifera carnica* ): Dependence on Individual Age and Function.” Journal of Insect Physiology 38, no. 6: 409–419. 10.1016/0022-1910(92)90117-V.

[ece371717-bib-0034] Crall, J. D. , J. J. Chang , R. L. Oppenheimer , and S. A. Combes . 2017. “Foraging in an Unsteady World: Bumblebee Flight Performance in Field Realistic Turbulence.” Interface Focus 7: 20160086. 10.1098/rsfs.2016.0086. 28163878 PMC5206605

[ece371717-bib-0036] De Landa, G. F. , D. Alberoni , L. Baffoni , et al. 2023. “The Gut Microbiome of Solitary Bees Is Mainly Affected by Pathogen Assemblage and Partially by Land Use.” Environmental Microbiomes 18: 38. 10.1186/s40793-023-00494-w. PMC10131457 37098635

[ece371717-bib-0037] Dolezal, A. G. , and A. L. Toth . 2018. “Feedbacks Between Nutrition and Disease in Honeybee Health.” Current Opinion in Insect Science 26: 114–119. 10.1016/j.cois.2018.02.006. 29764650

[ece371717-bib-0038] Donkersley, P. , G. Rhodes , R. W. Pickup , K. C. Jones , and K. Wilson . 2018. “Bacterial Communities Associated With Honeybee Food Stores Are Correlated With Land Use.” Ecology and Evolution 8, no. 10: 4743–4756. 10.1002/ece3.3999. 29876054 PMC5980251

[ece371717-bib-0039] du Rand, E. E. , C. Stutzer , H. Human , C. W. W. Pirk , and S. W. Nicolson . 2020. “Antibiotic Treatment Impairs Protein Digestion in the Honeybee, *Apis mellifera* .” Apidologie 51: 94–106. 10.1007/s13592-019-00718-4.

[ece371717-bib-0040] Encinas‐Viso, F. , J. Bovill , D. E. Albrecht , et al. 2022. “Pollen DNA Metabarcoding Reveals Cryptic Diversity and High Spatial Turnover in Alpine Plant‐Pollinator Networks.” Molecular Ecology 32, no. 23: 6377–6393. 10.1111/mec.16682. 36065738

[ece371717-bib-0041] Engel, P. , W. K. Kwong , Q. McFrederick , et al. 2016. “The Bee Microbiome: Impact on Bee Health and Model for Evolution and Ecology of Host‐Microbe Interactions.” MBio 7, no. 2: e02164‐15. 10.1128/mBio.02164-15. 27118586 PMC4850275

[ece371717-bib-0042] Engel, P. , V. G. Martinson , and N. A. Moran . 2012. “Functional Diversity Within the Simple Gut Microbiota of the Honeybee.” Proceedings of the National Academy of Sciences of the United States of America 109, no. 27: 11002–11007. 10.1073/pnas.1202970109. 22711827 PMC3390884

[ece371717-bib-0043] Escalas, A. , J. C. Auguet , A. Avouac , et al. 2022. “Shift and Homogenization of Gut Microbiome During Invasion in Marine Fishes.” Animal Microbiome 4: 37. 10.1186/s42523-022-00181-0. 35659312 PMC9167558

[ece371717-bib-0044] Estaki, M. , L. Jiang , N. A. Bokulich , et al. 2020. “QIIME 2 Enables Comprehensive End‐To‐End Analysis of Diverse Microbiome Data and Comparative Studies With Publicly Available Data.” Current Protocols in Bioinformatics 70, no. 1: e100. 10.1002/cpbi.100. 32343490 PMC9285460

[ece371717-bib-0045] Fick, S. E. , and R. J. Hijmans . 2017. “WorldClim 2: New 1‐Km Spatial Resolution Climate Surfaces for Global Land Areas.” International Journal of Climatology 37, no. 12: 4302–4315. 10.1002/joc.5086.

[ece371717-bib-0046] Fontaine, S. S. , and K. D. Kohl . 2020. “Gut Microbiota of Invasive Bullfrog Tadpoles Responds More Rapidly to Temperature Than a Noninvasive Congener.” Molecular Ecology 29, no. 13: 2449–2462. 10.1111/mec.15487. 32463954

[ece371717-bib-0047] Ghisbain, G. , M. Gérard , T. J. Wood , H. M. Hines , and D. Michez . 2021. “Expanding Insect Pollinators in the Anthropocene.” Biological Reviews 96, no. 6: 2755–2770. 10.1111/brv.12777. 34288353 PMC9292488

[ece371717-bib-0048] Goulson, D. 2003. “Effects of Introduced Bees on Native Ecosystems.” Annual Review of Ecology, Evolution, and Systematics 34: 1–26. 10.1146/annurev.ecolsys.34.011802.132355.

[ece371717-bib-0049] Goulson, D. , E. Nicholls , C. Botias , et al. 2015. “Bee Declines Driven by Combined Stress From Parasites, Pesticides, and Lack of Flowers.” Science 347, no. 6229: 1255957. 10.1126/science.1255957. 25721506

[ece371717-bib-0050] Hammer, T. J. , E. Le , A. N. Martin , and N. A. Moran . 2021. “The Gut Microbiota of Bumblebees.” Insectes Sociaux 68, no. 4: 287–301. 10.1007/s00040-021-00837-1. 35342195 PMC8956082

[ece371717-bib-0051] Han, L. , Z. M. Chang , C. S. Ren , et al. 2024. “Colony Performance of Three Native Bumblebee Species From South China and Association With Their Gut Microbiome.” Insect Science 31, no. 6: 1960–1983. 10.1111/1744-7917.13351. 38516802 PMC11632300

[ece371717-bib-0052] Hanley, M. E. , and D. Goulson . 2003. “Introduced Weeds Pollinated by Introduced Bees: Cause or Effect?” Weed Biology and Management 3, no. 4: 204–212. 10.1046/j.1444-6162.2003.00108.x.

[ece371717-bib-0053] Hawkins, J. , N. de Vere , A. Griffith , et al. 2015. “Using DNA Metabarcoding to Identify the Floral Composition of Honey: A New Tool for Investigating Honeybee Foraging Preferences.” PLoS One 10, no. 8: e0134735. 10.1371/journal.pone.0134735. 26308362 PMC4550469

[ece371717-bib-0055] Himler, A. G. , T. Adachi‐Hagimori , J. E. Bergen , et al. 2011. “Rapid Spread of a Bacterial Symbiont in an Invasive Whitefly Is Driven by Fitness Benefits and Female Bias.” Science 332, no. 6026: 254–256. 10.1126/science.1199410. 21474763

[ece371717-bib-0056] Hingston, A. B. 2006a. “Is the Exotic Bumblebee *Bombus terrestris* Really Invading Tasmanian Native Vegetation?” Journal of Insect Conservation 10, no. 3: 289–293. 10.1007/s10841-006-6711-7.

[ece371717-bib-0057] Hingston, A. B. 2006b. “Is the Introduced Bumblebee (*Bombus terrestris*) Assisting the Naturalization of the *Agapanthus praecox ssp. orientalis* in Tasmania?” Ecological Management & Restoration 7, no. 3: 236–240. 10.1111/j.1442-8903.2006.312_7.x.

[ece371717-bib-0058] Hingston, A. B. 2007. “The Potential Impact of the Large Earth Bumblebee ‘*Bombus terrestris*’ (Apidae) on the Australian Mainland: Lessons From Tasmania.” Victorian Naturalist 124, no. 2: 110–117. https://biostar.org/reference/236356.

[ece371717-bib-0059] Hingston, A. B. , J. O. N. Marsden‐Smedley , D. A. Driscoll , et al. 2002. “Extent of Invasion of Tasmanian Native Vegetation by the Exotic Bumblebee *Bombus terrestris* (Apoidea: Apidea).” Austral Ecology 27, no. 2: 162–172. 10.1046/j.1442-9993.2002.01179.x.

[ece371717-bib-0060] Hingston, A. B. , and P. B. McQuillan . 1998. “Nectar Robbing in *Epacris impressa* (Epacridaceae) by the Recently Introduced Bumblebee *Bombus terrestris* (Apidae) in Tasmania.” Victorian Naturalist 115: 116–119. https://biostar.org/reference/246068.

[ece371717-bib-0061] Hingston, A. B. , and P. B. McQuillan . 1999. “Displacement of Tasmanian Native Megachilid Bees by the Recently Introduced Bumblebee *Bombus terrestris* (Linnaeus, 1758) (Hymenoptera: Apidae).” Australian Journal of Zoology 47, no. 1: 59–65. 10.1071/ZO98016.

[ece371717-bib-0062] Hingston, A. B. , and S. Wotherspoon . 2017. “Introduced Social Bees Reduce Nectar Availability During the Breeding Season of the Swift Parrot (*Lathamus discolor*).” Pacific Conservation Biology 23, no. 1: 52–62. 10.1071/PC16025.

[ece371717-bib-0063] Hülsmann, M. , H. von Wehrden , A. Klein , et al. 2015. “Plant Diversity and Composition Compensate for Negative Effects of Urbanisation on Foraging Bumble Bees.” Apidologie 46: 760–770. 10.1007/s13592-015-0366-x.

[ece371717-bib-0064] Iovane, M. , A. Cirillo , L. G. Izzo , C. di Vaio , and G. Aronne . 2022. “High Temperature and Humidity Affect Pollen Viability and Longevity in *Olea europaea* L.” Agronomy 12, no. 1: 1. 10.3390/agronomy12010001.

[ece371717-bib-0065] Jackson, J. M. , M. L. Pimsler , K. J. Oyen , et al. 2018. “Distance, Elevation and Environment as Drivers of Diversity and Divergence in Bumble Bees Across Latitude and Altitude.” Molecular Ecology 27, no. 14: 2926–2942. 10.1111/mec.14735. 29862596

[ece371717-bib-0066] Jackson, J. M. , M. L. Pimsler , K. J. Oyen , J. P. Strange , M. E. Dillon , and J. D. Lozier . 2020. “Local Adaptation Across a Complex Bioclimatic Landscape in Two Montane Bumble Bee Species.” Molecular Ecology 29, no. 5: 920–939. 10.1111/mec.15376. 32031739

[ece371717-bib-0067] Kakumanu, M. L. , A. M. Reeves , T. D. Anderson , et al. 2016. “Honeybee Gut Microbiome Is Altered by In‐Hive Pesticide Exposures.” Frontiers in Microbiology 7: 1255. 10.3389/fmicb.2016.01255. 27579024 PMC4985556

[ece371717-bib-0068] Kantsa, A. , R. A. Raguso , T. Lekkas , O. I. Kalantzi , and T. Petanidou . 2019. “Floral Volatiles and Visitors: A Meta‐Network of Associations in a Natural Community.” Journal of Ecology 107: 2574–2586. 10.1111/1365-2745.13197.

[ece371717-bib-0069] Kardum Hjort, C. , J. R. Paris , H. G. Smith , and R. Y. Dudaniec . 2024. “Selection Despite Low Genetic Diversity and High Gene Flow in a Rapid Island Invasion of the Bumblebee, *Bombus terrestris* .” Molecular Ecology 33, no. 2: e17212. 10.1111/mec.17212. 37990959

[ece371717-bib-0070] Kardum Hjort, C. , H. G. Smith , A. P. Allen , and R. Y. Dudaniec . 2023. “Morphological Variation in Bumblebees ( *Bombus terrestris* ) (Hymenoptera: *Apidae*) After Three Decades of an Island Invasion.” Journal of Insect Science 23, no. 1: 10. 10.1093/jisesa/iead006. PMC9972831 36856678

[ece371717-bib-0071] Katoh, K. , K. Misawa , K. I. Kuma , and T. Miyata . 2002. “MAFFT: A Novel Method for Rapid Multiple Sequence Alignment Based on Fast Fourier Transform.” Nucleic Acids Research 30, no. 14: 3059–3066. 10.1093/nar/gkf436. 12136088 PMC135756

[ece371717-bib-0072] Keller, A. , N. Danner , G. Grimmer , et al. 2015. “Evaluating Multiplexed Next‐Generation Sequencing as a Method in Palynology for Mixed Pollen Samples.” Plant Biology 17, no. 2: 558–566. 10.1111/plb.12251. 25270225

[ece371717-bib-0073] Keller, A. , Q. S. McFrederick , P. Dharampal , et al. 2021. “(More Than) Hitchhikers Through the Network: The Shared Microbiome of Bees and Flowers.” Current Opinion in Insect Science 44: 8–15. 10.1016/j.cois.2020.09.007. 32992041

[ece371717-bib-0074] Kešnerová, L. , O. Emery , M. Troilo , J. Liberti , B. Erkosar , and P. Engel . 2020. “Gut Microbiota Structure Differs Between Honeybees in Winter and Summer.” ISME Journal 14, no. 3: 801–814. 10.1038/s41396-019-0568-8. 31836840 PMC7031341

[ece371717-bib-0075] Key to Tasmanian Vascular Plants . 2023. “University of Tasmania.” https://www.utas.edu.au/dicotkey/dicotkey/key.htm.

[ece371717-bib-0076] Koot, E. M. , M. Morgan‐Richards , and S. A. Trewick . 2022. “Climate Change and Alpine‐Adapted Insects: Modelling Environmental Envelopes of a Grasshopper Radiation.” Royal Society Open Science 9: 211596. 10.1098/rsos.211596. 35316945 PMC8889178

[ece371717-bib-0079] Kwong, W. K. , A. L. Mancenido , and N. A. Moran . 2017. “Immune System Stimulation by the Native Gut Microbiota of Honeybees.” Royal Society Open Science 4, no. 2: 170003. 10.1098/rsos.170003. 28386455 PMC5367273

[ece371717-bib-0080] Kwong, W. K. , L. A. Medina , H. Koch , et al. 2017. “Dynamic Microbiome Evolution in Social Bees.” Science Advances 3: e1600513. 10.1126/sciadv.1600513. 28435856 PMC5371421

[ece371717-bib-0081] Kwong, W. K. , and N. A. Moran . 2016. “Gut Microbial Communities of Social Bees.” Nature Reviews Microbiology 14: 374–384. 10.1038/nrmicro.2016.43. 27140688 PMC5648345

[ece371717-bib-0082] Lawson, D. A. , and S. A. Rands . 2019. “The Effects of Rainfall on Plant–Pollinator Interactions.” Arthropod‐Plant Interactions 13: 561–569. 10.1007/s11829-019-09686-z.

[ece371717-bib-0083] Lee, F. J. , K. I. Miller , J. B. McKinlay , et al. 2018. “Differential Carbohydrate Utilization and Organic Acid Production by Honeybee Symbionts.” FEMS Microbiology Ecology 94, no. 8: fiy113. 10.1093/femsec/fiy113. 29878200

[ece371717-bib-0084] Lee, F. J. , D. B. Rusch , F. J. Stewart , H. R. Mattila , and I. L. G. Newton . 2015. “Saccharide Breakdown and Fermentation by the Honeybee Gut Microbiome.” Environmental Microbiology 17, no. 3: 796–815. 10.1111/1462-2920.12526. 24905222

[ece371717-bib-0085] Lim, H. C. , C.‐C. Chu , M. J. Seufferheld , and S. A. Cameron . 2015. “Deep Sequencing and Ecological Characterization of Gut Microbial Communities of Diverse Bumble Bee Species.” PLoS One 10: e0118566. 10.1371/journal.pone.0118566. 25768110 PMC4359114

[ece371717-bib-0087] Lowenstein, D. M. , K. C. Matteson , and E. S. Minor . 2019. “Evaluating the Dependence of Urban Pollinators on Ornamental, Non‐Native, and ‘Weedy’ Floral Resources.” Urban Ecosystems 22, no. 1: 293–302. 10.1007/s11252-018-0817-z.

[ece371717-bib-0088] Lowenstein, D. M. , and E. S. Minor . 2016. “Diversity in Flowering Plants and Their Characteristics: Integrating Humans as a Driver of Urban Floral Resources.” Urban Ecosystems 19: 1735–1748. 10.1007/s11252-016-0563-z.

[ece371717-bib-0089] Lu, M. , J. Hulcr , and J. Sun . 2016. “The Role of Symbiotic Microbes in Insect Invasions.” Annual Review of Ecology, Evolution, and Systematics 47: 487–505. 10.1146/annurev-ecolsys-121415-032050.

[ece371717-bib-0090] Lucas, A. , O. Bodger , B. J. Brosi , et al. 2018. “Generalisation and Specialisation in Hoverfly (Syrphidae) Grassland Pollen Transport Networks Revealed by DNA Metabarcoding.” Journal of Animal Ecology 87, no. 4: 1008–1021. 10.1111/1365-2656.12828. 29658115 PMC6032873

[ece371717-bib-0091] Macfarlane, R. P. , and L. Gurr . 1995. “Distribution of Bumblebees in New Zealand.” New Zealand Entomologist 18, no. 1: 29–36. 10.1080/00779962.1995.9721999.

[ece371717-bib-0092] Manfredini, F. , M. Arbetman , and A. L. Toth . 2019. “A Potential Role for Phenotypic Plasticity in Invasions and Declines in Social Insects.” Fronters in Ecology and Evolution 7: 375. 10.3389/fevo.2019.00375.

[ece371717-bib-0093] Manning, R. 2001. “Fatty Acids in Pollen: A Review of Their Importance for Honeybees.” Bee World 82, no. 2: 60–75. 10.1080/0005772X.2001.11099504.

[ece371717-bib-0094] Martin, M. 2011. “Cutadapt Removes Adapter Sequences From High‐Throughput Sequencing Reads.” EMBnet. Journal 17, no. 1: 10–12. 10.14806/ej.17.1.200.

[ece371717-bib-0095] Martinez Arbizu, P. 2020. “Pairwise Adonis: Pairwise Multilevel Comparison Using Adonis.” R Package, Version 0.4. https://github.com/pmartinezarbizu/pairwiseAdonis.

[ece371717-bib-0096] Martinson, V. G. , B. N. Danforth , R. L. Minckley , et al. 2011. “A Simple and Distinctive Microbiota Associated With Honeybees and Bumblebees.” Molecular Ecology 20, no. 3: 619–628. 10.1111/j.1365-294X.2010.04959.x. 21175905

[ece371717-bib-0097] Matteson, K. C. , and G. A. Langellotto . 2009. “Bumble Bee Abundance in New York City Community Gardens: Implications for Urban Agriculture.” Cities and the Environment 2, no. 1: 5.

[ece371717-bib-0098] McFrederick, Q. S. , and S. M. Rehan . 2022. “Wild Bee Pollen Usage and Microbial Communities Co‐Vary Across Landscapes.” Microbial Ecology 77, no. 2: 513–522. 10.1007/s00248-018-1232-y. 30069710

[ece371717-bib-0099] McMurdie, P. J. , and S. Holmes . 2013. “Phyloseq: An R Package for Reproducible Interactive Analysis and Graphics of Microbiome Census Data.” Public Library of Science 8, no. 4: e61217. 10.1371/journal.pone.0061217. PMC3632530 23630581

[ece371717-bib-0100] Milla, L. , A. Schmidt‐Lebuhn , J. Bovill , et al. 2022. “Monitoring of Honey Bee Floral Resources With Pollen DNA Metabarcoding as a Complementary Tool to Vegetation Surveys.” Ecological Solutions and Evidence 3, no. 1: e12120. 10.1002/2668-8319.12120.

[ece371717-bib-0101] Motta, E. V. S. , and N. A. Moran . 2024. “The Honeybee Microbiota and Its Impact on Health and Disease.” Nature Reviews Microbiology 22, no. 3: 122–137. 10.1038/s41579-023-00990-3. 38049554 PMC10998682

[ece371717-bib-0102] Mountcastle, A. M. , S. Ravi , and S. A. Combes . 2015. “Nectar vs. Pollen Loading Affects the Tradeoff Between Flight Stability and Maneuverability in Bumblebees.” Proceedings of the National Academy of Sciences 112, no. 33: 10527–10532. 10.1073/pnas.1506126112. PMC4547240 26240364

[ece371717-bib-0103] Naug, D. 2009. “Nutritional Stress due to Habitat Loss May Explain Recent Honeybee Colony Collapses.” Biological Conservation 142, no. 10: 2369–2372. 10.1016/j.biocon.2009.04.007.

[ece371717-bib-0104] O'Connell, M. , Z. Jordan , E. McGilvray , et al. 2021. “Reap What You Sow: Local Plant Composition Mediates Bumblebee Foraging Patterns Within Urban Garden Landscapes.” Urban Ecosystems 24, no. 5: 391–404. 10.1007/s11252-020-01043-w.

[ece371717-bib-0105] Oksanen, J. , F. G. Blanchet , and M. Friendly . 2024. “Package ‘vegan.’” Community Ecology Package, Version 2.6‐4. https://cran.r‐project.org/web/packages/vegan/vegan.pdf.

[ece371717-bib-0106] Otterstatter, M. C. , and J. D. Thomson . 2008. “Does Pathogen Spillover From Commercially Reared Bumble Bees Threaten Wild Pollinators?” PLoS One 3: e2771. 10.1371/journal.pone.0002771. 18648661 PMC2464710

[ece371717-bib-0107] Page, M. L. , and N. M. Williams . 2023. “Evidence of Exploitative Competition Between Honey Bees and Native Bees in Two California Landscapes.” Journal of Animal Ecology 92: 1802–1814. 10.1111/1365-2656.13973. 37386764

[ece371717-bib-0108] Pinheiro, J. , D. Bates , S. DebRoy , D. Sarkar , and R Core Team . 2024. “nlme: Linear and Nonlinear Mixed Effects Models.” Version 3.1‐166. https://cran.r‐project.org/web/packages/nlme/nlme.pdf.

[ece371717-bib-0109] Pornon, A. , C. Andalo , M. Burrus , and N. Escaravage . 2017. “DNA Metabarcoding Data Unveils Invisible Pollination Networks.” Scientific Reports 7: 16828. 10.1038/s41598-017-16785-5. 29203872 PMC5715002

[ece371717-bib-0111] Price, M. N. , P. S. Dehal , and A. P. Arkin . 2010. “FastTree 2 – Approximately Maximum‐Likelihood Trees for Large Alignments.” Public Library of Science 5, no. 3: e9490. 10.1371/journal.pone.0009490. PMC2835736 20224823

[ece371717-bib-0112] Quast, C. , E. Pruesse , P. Yilmaz , et al. 2012. “The SILVA Ribosomal RNA Gene Database Project: Improved Data Processing and Web‐Based Tools.” Nucleic Acids Research 41, no. Database issue: D590–D596. 10.1093/nar/gks1219. 23193283 PMC3531112

[ece371717-bib-0113] R Core Team . 2024. R: A Language and Environment for Statistical Computing. R Foundation for Statistical Computing. [Methodology Reference]. https://www.R‐project.org/.

[ece371717-bib-0114] Ravi, S. , D. Kolomenskiy , T. Engels , et al. 2016. “Bumblebees Minimize Control Challenges by Combining Active and Passive Modes in Unsteady Winds.” Scientific Reports 6: 35043. 10.1038/srep35043. 27752047 PMC5067513

[ece371717-bib-0115] Robeson, M. S. , D. R. O'Rourke , B. D. Kaehler , et al. 2021. “RESCRIPt: Reproducible Sequence Taxonomy Reference Database Management.” PLoS Computational Biology 17, no. 11: e1009581. 10.1371/journal.pcbi.1009581. 34748542 PMC8601625

[ece371717-bib-0116] Rosso, F. , V. Tagliapietra , D. Albanese , et al. 2018. “Reduced Diversity of Gut Microbiota in Two *Aedes* Mosquitoes Species in Areas of Recent Invasion.” Scientific Reports 8: 16091. 10.1038/s41598-018-34640-z. 30382151 PMC6208342

[ece371717-bib-0117] Roulston, T. H. , and S. L. Buchmann . 2000. “A Phylogenetic Reconsideration of the Pollen Starch‐Pollination Correlation.” Evolutionary Ecology Research 2, no. 5: 627–643.

[ece371717-bib-0120] Russo, L. 2016. “Positive and Negative Impacts of Non‐Native Bee Species Around the World.” Insects 7, no. 4: 69. 10.3390/insects7040069. 27916802 PMC5198217

[ece371717-bib-0122] Scarth, P. 2013. Vegetation Height and Structure ‐ Derived From ALOS‐1 PALSAR, Landsat and ICESat/GLAS, Australia Coverage. Terrestrial Ecosystem Research Network (TERN). https://portal.tern.org.au/metadata/TERN/de1c2fef‐b129‐485e‐9042‐8b22ee616e66.

[ece371717-bib-0123] Schmid‐Hempel, P. , R. Schmid‐Hempel , P. C. Brunner , O. D. Seeman , and G. R. Allen . 2007. “Invasion Success of the Bumblebee, *Bombus terrestris* , Despite a Drastic Genetic Bottleneck.” Heredity 99, no. 4: 414–422. 10.1038/sj.hdy.6801017. 17551520

[ece371717-bib-0124] Semmens, T. D. , E. Turner , and R. Buttermore . 1993. “ *Bombus terrestris* (L.) (Hymenoptera: Apidae) Now Established in Tasmania.” Australian Journal of Entomology 32, no. 4: 346. 10.1111/j.1440-6055.1993.tb00598.x.

[ece371717-bib-0125] Singh, T. , M. M. Bhat , and M. A. Khan . 2009. “Insect Adaptations to Changing Environments – Temperature and Humidity.” International Journal of Industrial Entomology 19, no. 1: 155–164. https://koreascience.kr/article/JAKO200933063804084.pdf.

[ece371717-bib-0126] Su, Q. , Q. Wang , X. Mu , et al. 2021. “Strain‐Level Analysis Reveals the Vertical Microbial Transmission During the Life Cycle of Bumblebee.” Microbiome 9, no. 1: 216. 10.1186/s40168-021-01163-1. 34732245 PMC8567698

[ece371717-bib-0129] Wright, G. A. , S. W. Nicolson , and S. Shafir . 2018. “Nutritional Physiology and Ecology of Honey Bees.” Annual Review of Entomology 63: 327–344. 10.1146/annurev-ento-020117-043423. 29029590

[ece371717-bib-0130] Yao, H. , J. Song , C. Liu , et al. 2010. “Use of ITS2 Region as the Universal DNA Barcode for Plants and Animals.” Public Library of Science 5, no. 10: e13102. 10.1371/journal.pone.0013102. PMC2948509 20957043

[ece371717-bib-0131] Yilmaz, P. , L. W. Parfrey , P. Yarza , et al. 2014. “The SILVA and ‘All‐Species Living Tree Project (LTP)’ Taxonomic Frameworks.” Nucleic Acids Research 42: D643–D648. 10.1093/nar/gkt1209. 24293649 PMC3965112

[ece371717-bib-0132] Yokota, S. C. , C. Broeckling , and H. S. A. Seshadri . 2024. “Pollen Foraging Preferences in Honeybees and the Nutrient Profiles of the Pollen.” Scientific Reports 14: 15028. 10.1038/s41598-024-65569-1. 38951538 PMC11217361

[ece371717-bib-0133] Zhang, Z. , Y. Guo , M. Zhuang , et al. 2023. “Potential Role of the Gut Microbiota of Bumblebee *Bombus pyrosoma* in Adaptation to High‐Altitude Habitats.” Frontiers in Microbiology 14: 1218560. 10.3389/fmicb.2023.1218560. 37601385 PMC10433375

[ece371717-bib-0135] Zheng, H. , J. Perreau , J. E. Powell , et al. 2019. “Division of Labor in Honeybee Gut Microbiota for Plant Polysaccharide Digestion.” Proceedings of the National Academy of Sciences of the United States of America 116, no. 51: 25909–25916. 10.1073/pnas.1916224116. 31776248 PMC6926048

[ece371717-bib-0136] Zhu, L. , Z. Zhang , H. Chen , et al. 2021. “Gut Microbiomes of Bigheaded Carps and Hybrids Provide Insights Into Invasion: A Hologenome Perspective.” Evolutionary Applications 14, no. 3: 735–745. 10.1111/eva.13152. 33767748 PMC7980309

