# Supplementary material for: Landscape‐Wide Metabarcoding Shows Interactions Among the Gut Microbiome and Pollen Diversity in the Invasive Bumblebee, Bombus terrestris
Source: Ecol Evol. 2025 Jul 7;15(7):e71717. doi: 10.1002/ece3.71717 (PMC12230354; doi:10.1002/ece3.71717)
Supplement: Supplementary file 1 — Appendix S1. [file ECE3-15-e71717-s001.docx]

**SUPPLEMENTARY MATERIAL**

**Landscape-wide metabarcoding shows interactions among the gut microbiome and pollen diversity in the invasive bumblebee, *Bombus terrestris***

Sabrina Haque^1^, Hasinika KAH Gamage^1,2^, Cecilia Kardum Hjort^1,5^, Fleur Ponton^1,2^, Francisco Encinas-Viso^4^, Ian T Paulsen^1,2,3^, Rachael Y Dudaniec^1^

*^1^School of Natural Sciences, Macquarie University, NSW 2109, Australia*

*^2^ARC Training Centre for Facilitated Advancement of Australia’s Bioactives, Macquarie University, NSW 2109, Australia*

*^3^ARC Centre of Excellence in Synthetic Biology, Macquarie University, NSW 2109, Australia*

*^4^Centre for Australian National Biodiversity Research, CSIRO, Black Mountain, Australian Capital Territory, Australia*

*^5^Department of Biology, Lund University, Lund, Sweden*

***Text S1. Field Sampling***

During the active summer flight period in February 2020, free-flying *B. terrestris* workers were opportunistically captured from each site over a two-week period. The bumblebees were caught using handheld entomological sweep nets and jars, and the sampling was limited to 90 minutes. Captured worker bees were individually placed into 5ml plastic tubes, stored in a battery-powered car refrigerator (~4°C) to induce chill coma. Subsequently, the bees were sexed (determined by presence of a stinger and/or corbicular pollen) and placed in a freezer (–18°C) for approximately 3 hours to induce euthanasia. Finally, the bees were preserved in 70% ethanol, following procedures outlined in Kardum Hjort et al. (2023; 2024).

***Text S2: Bumblebee gut dissection and gut bacterial DNA extraction***

Prior to dissection, each *B. terrestris* individual was rinsed with 70% ethanol, placed on a small sterile petri dish, and immersed in sterile phosphate buffer solution (1x PBS, 137 mM NaCl; 2.7 mM KCl; 10 mM NaH_2_PO_4_; 1.8 mM KH_2_PO_4_). Dissections to separate the midgut and the hindgut from the body were carried out under a binocular stereo microscope (Motic SMZ 1711) with sterile forceps. The intact mid and hindgut were stored in 600μl 1xPBS. DNA was extracted from *B. terrestris* gut samples using a modification of the DNeasy Blood and Tissue Kit protocol (Qiagen). For pre-treatment of Gram-positive bacteria prior to the extraction, enzymatic lysis buffer (20 mM Tris-Cl, pH=8.0; 2mM Na_2_EDTA; 1.2% Triton X-100) was supplemented (20 mg ml-1) with lysozyme (Thermo Fisher Scientific), and 180 μl of this lysozyme-supplemented enzymatic lysis buffer was added to each gut sample. Glass beads (0.1 mm, Benchmark Scientific) were added to the samples and incubated on the heating block at 37℃ for 45 minutes. The samples were homogenised using a Tissue Lyser II (Qiagen) for five minutes at 30Hz and then samples were incubated again at 37℃ for 45 minutes. After lysis activity, 200μl of Buffer AL and 25μl of Proteinase-K (Ambion) was added to the samples and incubated at 56℃ for 30 minutes. The rest of the steps for the DNA extraction were carried out based on the manufacturer’s protocol. DNA concentration was measured using a Qubit 4 Fluorometer using dsDNA High-Sensitivity (HS) Assay Kit (Invitrogen).

***Text S3. DNA metabarcoding library preparation and sequencing***

16S V4 region amplicon sequencing was undertaken by the Ramaciotti Centre for Genomics (University of New South Wales, Sydney, Australia). The gene-specific full-length primer sequences, targeting the V4 region were:

16S Amplicon PCR Forward Primer = 5’ TCGTCGGCAGCGTCAGATGTGTATAAGAGACAGCCTACGGGNGGCWGCAG and

16S Amplicon PCR Reverse Primer = 5’ GTCTCGTGGGCTCGGAGATGTGTATAAGAGACAGGACTACHVGGGTATCTAATCC.

The Illumina overhang adapter sequences added to the locus‐specific primer for the target region: Forward overhang: 5’ TCGTCGGCAGCGTCAGATGTGTATAAGAGACAG‐[locus specific sequence] and Reverse overhang: 5’GTCTCGTGGGCTCGGAGATGTGTATAAGAGACAG‐[locus specific sequence].

To amplify the 16S gene, PCR conditions comprised an initial denaturation step at 95°C for 3 minutes, followed by 25 cycles of 95°C for 30 seconds, 55°C annealing for 30 seconds, 72°C extension for 30 seconds, and a final extension at 72°C for 5 minutes. Following this PCR, purification of the PCR products was executed using AMPure XP beads to eliminate free primers and primer dimers from the amplicons. Subsequently, a dual indexing process and attachment of Illumina sequencing adapters were conducted through an index PCR, utilizing the Nextera XT Index Kit (FC-131-1001). The conditions for this PCR began with 95°C for 3 minutes which was followed by 8 cycles of 95°C for 30 seconds, 55°C annealing for 30 seconds, 72°C for 30 seconds, and a final extension at 72°C for 5 minutes. A secondary PCR clean-up step utilizing AMPure XP beads was performed before the library underwent final quantification and validation. A 1:50 dilution of the ultimate library was ran on a Bioanalyzer DNA 1000 chip to confirm the size. The pooled libraries were subsequently denatured with NaOH, diluted with hybridization buffer, and heat-denatured before paired-end 2x250 sequencing on the Illumina MiSeq platform.

***Text S4. Pollen removal from bees, DNA extraction and ITS2 metabarcoding***

***Pollen removal***

The sample tubes containing *B. terrestris* workers and 70% ethanol were gently shaken to

dislodge any corbicular pollen or grains attached to the bees and the bees were moved to a

sterile petri dish. The collected pollen was transferred to a new Eppendorf tube, while the bee was returned to its original tube with ethanol. The pollen tube was centrifuged at 10000 rpm for three minutes. The ethanol supernatant was removed, ensuring removal of other debris while keeping the pollen pellet intact. The pollen pellet was washed in 200μl of DNase/RNase-free water (Invitrogen, Life Technologies) and the contents were transferred into a sterile 2ml ‘master tube’, which was centrifuged again at 10000 rpm for five minutes and the supernatant was removed. The procedure was repeated for each bee, with the pollen from each bee of the same site pooled within the master tube, which was then stored at -30℃ until DNA extraction.

***DNA extraction***

The pooled pollen samples from each site were extracted for DNA using a modified protocol with the NucleoSpin Food Kit (Macherey Nagel). The CF (lysis) buffer was heated in a heating block for 10 minutes at 65℃, and 1 ml of the heated buffer was added to each pollen sample. Glass and zirconium oxide beads (2 mm, Lysing Matrix H; MP Biomedicals) were added to each sample tube, which was then homogenised using a Tissue Lyser II (Qiagen) for three minutes at 20 Hz. Proteinase-K (30μl) was then added, and samples were incubated at 65℃ in for 1.5 hours. The subsequent steps of the pollen DNA extraction adhered to the manufacturer’s protocol. Quantification of the extracted DNA was conducted using a Qubit 4 Fluorometer with the dsDNA HS Assay Kit (Invitrogen).

***PCR***

PCR was conducted on the extracted pollen DNA samples to amplify the ITS2 region

with 12.5μl of AmpliTaq Gold 360 MasterMix (Life Technologies), 0.5 μl of forward primer S2F (5'-ATGCGATACTTGGTGTGAAT-3') (0.2 μM), 0.5 μl of reverse primer S3R (5'-

GACGCTTCTCCAGACTACAAT-3') (0.2 μM), 8.5 μl of DNase/RNase-free water (Invitrogen, Life Technologies), and 3μl of pollen DNA sample (Chen et al., 2010). The PCR protocol consisted of an initial denaturation at 94℃ for 5 minutes, followed by 30 cycles of 94℃ for 30 seconds, 56℃ annealing for 30 seconds, 72℃ extension for 45 seconds and a final extension at 72℃ for 10 minutes. At the Ramaciotti Centre for Genomics (UNSW, Sydney, Australia), PCR products were subjected to purification, followed by a secondary PCR clean-up, library preparation and 2x250 bp paired-end sequencing was performed on an Illumina MiSeq platform (Text S3).

**Table S1.** Pearson’s correlation matrix for all environmental variables.  Mean seasonal precipitation was excluded from further analyses due to its notable strong positive correlation (r $\geq$ 0.7) with mean annual precipitation (r = 0.71). The final six selected environmental variables are in bold. Abbreviations: Annual Temp = Mean annual temperature ($℃)$, Annual Precip = Mean annual precipitation (mm), Seasonal Precip = Precipitation seasonality (mm), Percent Pasture = Percentage of pasture (%), Veg Height = Height of vegetation (mm), Percent Urban = Percentage of urbanisation (%) and Wind = Average velocity of summer wind (m/s).

|  | **Annual Temp** | **Annual Precip** | Seasonal Precip | **Percent Pasture** | **Veg Height** | **Percent Urban** | **Wind** |
| --- | --- | --- | --- | --- | --- | --- | --- |
| **Annual Temp** | 1 | -0.01 | 0.37 | -0.06 | -0.05 | 0.28 | 0.63 |
| **Annual Precip** | -0.01 | 1 | 0.71 | -0.41 | 0.47 | -0.19 | 0.23 |
| Seasonal Precip | 0.37 | **0.71** | 1 | -0.35 | 0.29 | -0.04 | 0.31 |
| **Percent Pasture** | -0.06 | -0.41 | -0.35 | 1 | -0.51 | -0.2 | -0.13 |
| **Veg Height** | -0.05 | 0.47 | 0.29 | -0.51 | 1 | 0.04 | 0.46 |
| **Percent Urban** | 0.28 | -0.19 | -0.04 | -0.2 | 0.04 | 1 | 0.25 |
| **Wind** | 0.63 | 0.23 | 0.31 | -0.13 | 0.46 | 0.25 | 1 |

**Table S2.** Total list of plant genera foraged by *B. terrestris* across Tasmania, identified via ITS2 sequencing of corbicular pollen. ‘Native’ = plant genera native to Tasmania, ‘Introduced’ = exotic plant genera that have been naturalised in Tasmania, ‘Both’ = plant genera consisting of both native and introduced species in Tasmania.

| **Introduced** | **Native** | **Both** |
| --- | --- | --- |
| Rubus | Eucalyptus | Plantago |
| Hypochaeris | Leptospermum | Solanum |
| Lupinus | Clematis | Veronica |
| Lotus | Correa | Borago |
| Raphanus | Myoporum | Melaleuca |
| Reseda |  | Sonchus |
| Sisymbrium |  | Epilobium |
| Pisum |  | Senecio |
| Trifolium |  | Acacia |
| Rosa |  | Erigeron |
| Digitalis |  |  |
| Osmanthus |  |  |
| Prunus |  |  |
| Brassica |  |  |
| Cirsium |  |  |
| Glycine Max |  |  |
| Circaea |  |  |
| Cichorium |  |  |
| Syzigium |  |  |
| Medicago |  |  |
| Sanguisorba |  |  |
| Cosmos |  |  |
| Centaurium |  |  |
| Vigna |  |  |
| Tropaeolum |  |  |
| Achillea |  |  |
| Anagalis |  |  |
| Kickxia |  |  |
| Leucanthemum |  |  |
| Arnoseris |  |  |
| Buddleja |  |  |
| Hydrangea |  |  |
| Reynoutria |  |  |
| Wisteria |  |  |
| Corymbia |  |  |

**Table S3.** Linear mixed-effect interactions between *B. terrestris* gut bacterial richness, pollen packet richness and environmental variables. Abbreviations: Bacterial_richness = Chao1 richness estimate of gut bacterial samples, Pollen_richness = Chao1 richness estimate of pollen samples, AT = Mean annual temperature (℃), AR = Mean annual precipitation (mm), PP = Percentage of pasture (%), VH = Height of vegetation (mm), PU = Percentage of urbanisation (%), WV = Average velocity of summer wind (m/s) and DF = Degrees of freedom. The asterisk (*) denotes a model with main effect and interaction.

| **Random effect = ~1\|Sites** | | | |
| --- | --- | --- | --- |
| **Fixed effect(s)** | **DF** | **t-value** | **p-value** |
| Bacterial_richness~AT | 12 | 0.20 | 0.85 |
| Bacterial_richness~AR | 12 | 0.99 | 0.34 |
| Bacterial_richness~PP | 12 | -1.07 | 0.30 |
| Bacterial_richness~PU | 12 | 0.55 | 0.60 |
| Bacterial_richness~VH | 12 | -0.31 | 0.76 |
| Bacterial_richness~WV | 12 | -1.18 | 0.26 |
| Bacterial_richness~AT*AR | 10 | -0.56 | 0.59 |
| Bacterial_richness~AT*PP | 10 | -0.28 | 0.79 |
| Bacterial_richness~AT*PU | 10 | 0.93 | 0.37 |
| Bacterial_richness~AT*VH | 10 | -1.29 | 0.22 |
| Bacterial_richness~AT*WV | 10 | -0.02 | 0.98 |
| Bacterial_richness~AR*PP | 10 | -0.92 | 0.38 |
| Bacterial_richness~AR*PU | 10 | 0.20 | 0.84 |
| Bacterial_richness~AR*VH | 10 | 1.07 | 0.31 |
| Bacterial_richness~AR*WV | 10 | -0.36 | 0.73 |
| Bacterial_richness~PP*PU | 10 | -0.89 | 0.39 |
| Bacterial_richness~PP*VH | 10 | -1.03 | 0.32 |
| Bacterial_richness~PP*WV | 10 | -0.06 | 0.95 |
| Bacterial_richness~PU*VH | 10 | -0.84 | 0.42 |
| Bacterial_richness~PU*WV | 10 | -0.54 | 0.60 |
| Bacterial_richness~VH*WV | 10 | -1.18 | 0.26 |
| Bacterial_richness~Pollen_richness | 12 | 0.16 | 0.87 |
| Bacterial_richness~Pollen_richness*AT | 10 | 0.13 | 0.90 |
| Bacterial_richness~Pollen_richness*AR | 10 | -1.39 | 0.19 |
| Bacterial_richness~Pollen_richness*PP | 10 | -0.11 | 0.91 |
| Bacterial_richness~Pollen_richness*PU | 10 | -1.32 | 0.21 |
| Bacterial_richness~Pollen_richness*VH | 10 | 0.91 | 0.38 |
| Bacterial_richness~Pollen_richness*WV | 10 | 0.86 | 0.41 |

**Table S4.** Environmental vectors and their correlation from NMDS ordination. Statistically significant (p $\leq$ 0.05) environmental variables and their r^2^ values are highlighted in bold (For corresponding NMDS ordination plots, see Figs. 4 and S2)

| *Environmental variables* | *r^2^* | *p* |
| --- | --- | --- |
| Mean annual temperature (AT) | 0.006 | 0.77 |
| **Mean annual precipitation (AR)** | **0.091** | **0.01** |
| **Percentage of pasture (PP)** | **0.090** | **0.02** |
| Percentage of urbanisation (PU) | 0.009 | 0.67 |
| Height of vegetation (VH) | 0.022 | 0.34 |
| Average summer wind velocity (WV) | 0.008 | 0.73 |

|  | S1 | S2 | S4 | S5 | S6 | S9 | S15 | S17 | S18 | S19 | S20 | S22 | S23 | S24 | S25 |
| --- | --- | --- | --- | --- | --- | --- | --- | --- | --- | --- | --- | --- | --- | --- | --- |
| S2 | 0.862 |  |  |  |  |  |  |  |  |  |  |  |  |  |  |
| S4 | 0.177 | 0.222 |  |  |  |  |  |  |  |  |  |  |  |  |  |
| S5 | **0.021** | **0.006** | 0.241 |  |  |  |  |  |  |  |  |  |  |  |  |
| S6 | 0.165 | 0.117 | 0.370 | 0.147 |  |  |  |  |  |  |  |  |  |  |  |
| S9 | **0.012** | **0.007** | **0.019** | 0.600 | **0.035** |  |  |  |  |  |  |  |  |  |  |
| S15 | 0.700 | 0.690 | 0.236 | **0.044** | 0.191 | **0.012** |  |  |  |  |  |  |  |  |  |
| S17 | 0.090 | 0.068 | 0.051 | 0.067 | **0.026** | **0.005** | 0.653 |  |  |  |  |  |  |  |  |
| S18 | 0.174 | 0.246 | 0.812 | 0.191 | 0.403 | **0.003** | 0.467 | 0.052 |  |  |  |  |  |  |  |
| S19 | 0.065 | 0.176 | 0.501 | 0.050 | 0.124 | **0.001** | 0.102 | **0.021** | 0.544 |  |  |  |  |  |  |
| S20 | **0.007** | **0.002** | 0.053 | 0.079 | **0.027** | **0.005** | **0.013** | **0.007** | 0.200 | 0.298 |  |  |  |  |  |
| S22 | 0.054 | 0.060 | **0.024** | **0.004** | **0.008** | 0.194 | **0.016** | **0.004** | **0.018** | **0.005** | **0.004** |  |  |  |  |
| S23 | 0.140 | **0.048** | 0.292 | 0.058 | 0.074 | **0.002** | 0.142 | **0.047** | 0.277 | 0.136 | 0.097 | **0.005** |  |  |  |
| S24 | 0.064 | 0.055 | **0.029** | **0.031** | **0.033** | **0.016** | 0.644 | 0.973 | **0.023** | **0.022** | **0.006** | **0.008** | **0.012** |  |  |
| S25 | 0.574 | 0.734 | 0.702 | **0.023** | 0.626 | **0.012** | 0.768 | 0.096 | 0.674 | 0.160 | **0.007** | **0.019** | 0.098 | 0.076 |  |
| S26 | 0.245 | 0.131 | 0.144 | 0.086 | 0.609 | **0.040** | 0.400 | 0.218 | 0.115 | **0.045** | **0.009** | **0.006** | **0.02** | 0.200 | 0.552 |

**Table S5.** Summary of pairwise PERMANOVA conducted to analyse significance of *B. terrestris* gut bacterial community composition between sites. Cells with p-values emphasized in bold indicates statistically significant sites (p $\leq$ 0.05)

|  | S1 | S15 | S17 | S18 | S19 | S2 | S20 | S22 | S23 | S24 | S25 | S26 | S4 | S5 | S6 |
| --- | --- | --- | --- | --- | --- | --- | --- | --- | --- | --- | --- | --- | --- | --- | --- |
| S15 | 1 |  |  |  |  |  |  |  |  |  |  |  |  |  |  |
| S17 | 1 | 1 |  |  |  |  |  |  |  |  |  |  |  |  |  |
| S18 | 1 | 1 | 1 |  |  |  |  |  |  |  |  |  |  |  |  |
| S19 | 1 | 1 | 1 | 1 |  |  |  |  |  |  |  |  |  |  |  |
| S2 | 1 | 1 | 1 | 1 | 1 |  |  |  |  |  |  |  |  |  |  |
| S20 | 1 | 1 | 1 | 1 | 1 | 1 |  |  |  |  |  |  |  |  |  |
| S22 | 1 | 1 | 1 | 1 | 1 | 1 | 1 |  |  |  |  |  |  |  |  |
| S23 | 1 | 1 | 1 | 1 | 1 | 0.083 | 0.913 | 0.979 |  |  |  |  |  |  |  |
| S24 | 1 | 1 | 1 | 1 | 1 | 1 | 1 | 1 | 1 |  |  |  |  |  |  |
| S25 | 1 | 1 | 1 | 1 | 1 | 0.684 | 1 | 1 | 1 | 1 |  |  |  |  |  |
| S26 | 1 | 1 | 1 | 1 | 1 | 1 | 1 | 1 | 1 | 1 | 1 |  |  |  |  |
| S4 | 1 | 1 | 1 | 1 | 1 | 1 | 1 | 1 | 0.182 | 1 | 1 | 1 |  |  |  |
| S5 | 1 | 1 | 1 | 1 | 1 | 1 | 1 | 1 | 0.351 | 1 | 1 | 1 | 1 |  |  |
| S6 | 1 | 1 | 1 | 1 | 1 | 1 | 1 | 1 | 1 | 1 | 1 | 1 | 1 | 1 |  |
| S9 | 1 | 1 | 1 | 1 | 1 | 1 | 1 | 1 | 1 | 1 | 1 | 1 | 1 | 1 | 1 |

**Table S6.** Summary of *t-test* conducted to analyse the significance of differences in Chao1 richness index of *B. terrestris* gut bacteria between sites. All sites are statistically insignificant (p > 0.05).

|  | S1 | S2 | S4 | S5 | S6 | S9 | S15 | S17 | S18 | S19 | S20 | S22 | S23 | S24 | S25 |
| --- | --- | --- | --- | --- | --- | --- | --- | --- | --- | --- | --- | --- | --- | --- | --- |
| S2 | 0.999 |  |  |  |  |  |  |  |  |  |  |  |  |  |  |
| S4 | 1 | 1 |  |  |  |  |  |  |  |  |  |  |  |  |  |
| S5 | 0.999 | 0.996 | 0.999 |  |  |  |  |  |  |  |  |  |  |  |  |
| S6 | 0.999 | 0.958 | 0.999 | 1 |  |  |  |  |  |  |  |  |  |  |  |
| S9 | 0.308 | 0.087 | 0.349 | 0.723 | 0.913 |  |  |  |  |  |  |  |  |  |  |
| S15 | 1 | 1 | 1 | 0.999 | 0.997 | 0.283 |  |  |  |  |  |  |  |  |  |
| S17 | 0.997 | 0.894 | 0.994 | 0.999 | 1 | 0.987 | 0.986 |  |  |  |  |  |  |  |  |
| S18 | 0.999 | 1 | 1 | 0.994 | 0.947 | 0.078 | 1 | 0.876 |  |  |  |  |  |  |  |
| S19 | 0.998 | 1 | 0.999 | 0.898 | 0.688 | **0.010** | 0.999 | 0.554 | 1 |  |  |  |  |  |  |
| S20 | 0.999 | 1 | 0.999 | 0.974 | 0.865 | **0.032** | 1 | 0.754 | 1 | 1 |  |  |  |  |  |
| S22 | 0.999 | 0.988 | 0.999 | 1 | 1 | 0.736 | 0.999 | 0.999 | 0.983 | 0.814 | 0.941 |  |  |  |  |
| S23 | 0.999 | 0.996 | 0.999 | 1 | 1 | 0.624 | 0.999 | 0.999 | 0.994 | 0.895 | 0.975 | 1 |  |  |  |
| S24 | 0.900 | 0.532 | 0.888 | 0.997 | 0.999 | 0.999 | 0.835 | 1 | 0.499 | 0.162 | 0.322 | 0.999 | 0.994 |  |  |
| S25 | 0.999 | 0.966 | 0.999 | 1 | 1 | 0.895 | 0.998 | 1 | 0.957 | 0.720 | 0.885 | 1 | 1 | 0.999 |  |
| S26 | 0.763 | 0.357 | 0.762 | 0.982 | 0.999 | 0.999 | 0.689 | 0.999 | 0.329 | 0.082 | 0.187 | 0.986 | 0.964 | 1 | 0.998 |

**Table S7.** Summary of ANOVA conducted to analyse the significance of differences in Shannon’s diversity index of *B. terrestris* gut bacteria between sites. Cells with p-values emphasized in bold shows statistically significant sites (p $\leq$ 0.05).


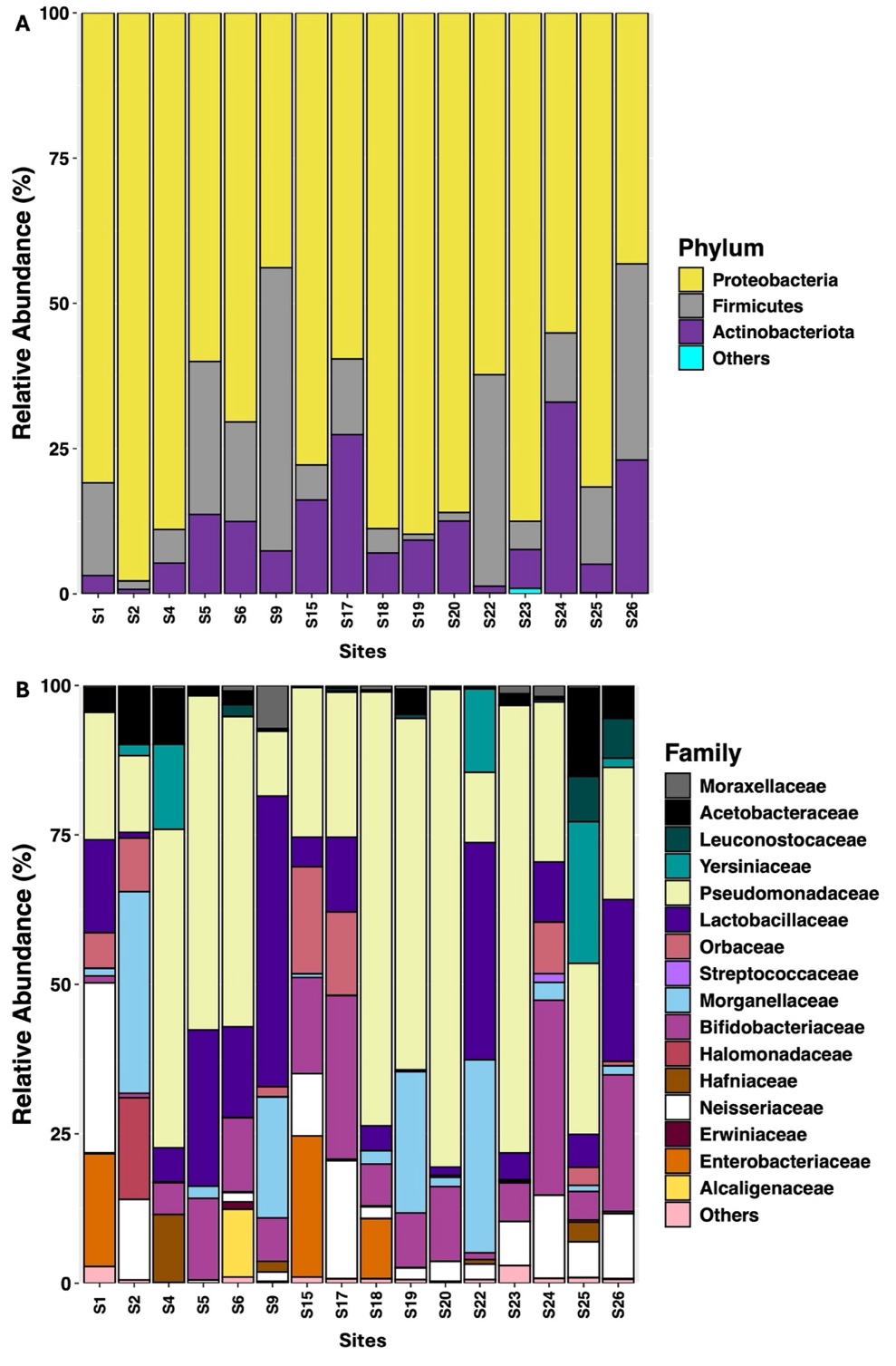


**Figure S1.** (A) Relative abundance of major gut bacterial phyla from *B. terrestris* per site. ‘Others’ indicate the relative abundance of all other bacterial phyla that contributed less than 1%. (B) Relative abundance of major gut bacterial families from *B. terrestris* per site. ‘Others’ indicate the relative abundance of all other bacterial families that contributed less than 1%.

**
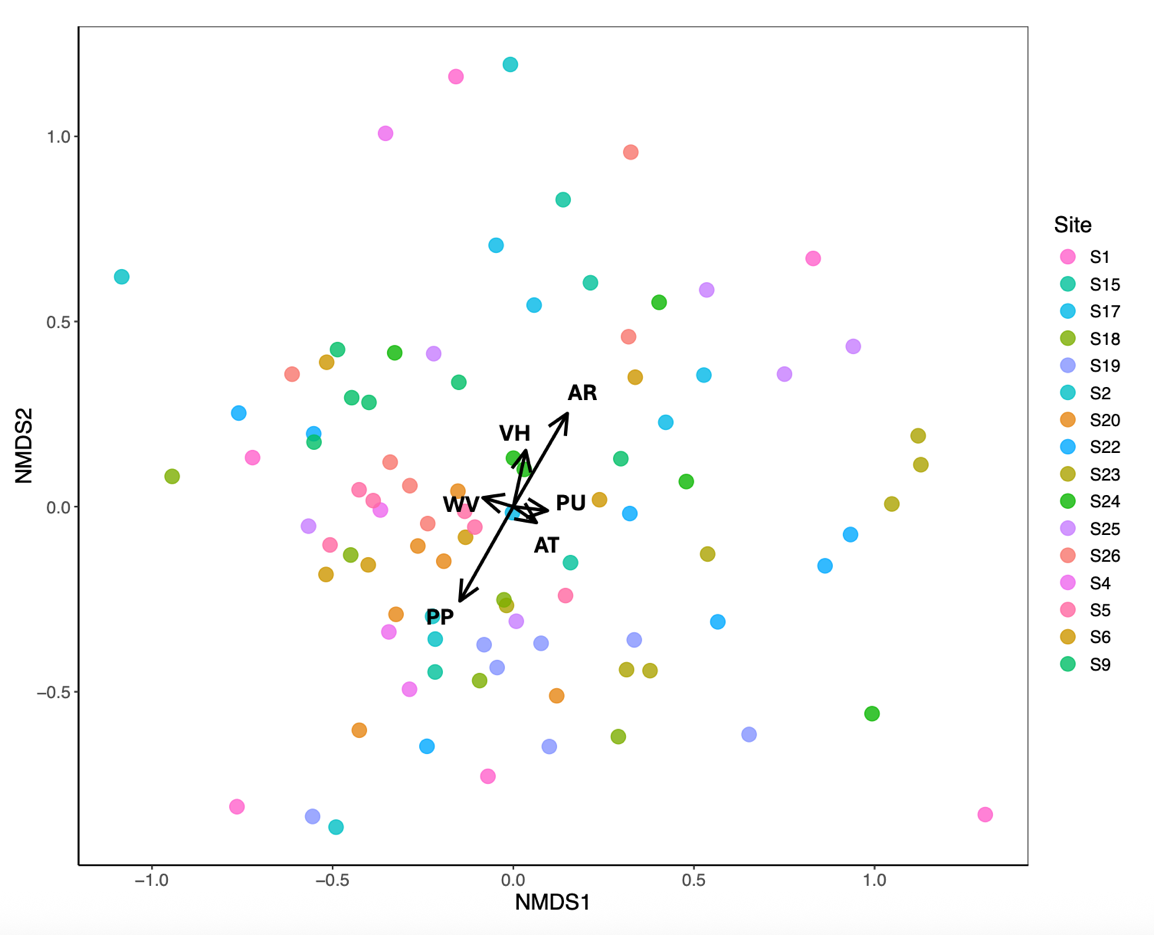
**

**Figure S2.** NMDS ordination of *B. terrestris* gut bacterial community based on Bray-Curtis dissimilarity of ASV abundance of individual samples. Stress = 0.20. Abbreviations: Abbreviations: AT = Mean annual temperature (°C), AR = Mean annual precipitation (mm), PP = Percentage of pasture (%), PU = Percentage of urbanisation (%), VH = Height of vegetation (mm), WV = Average summer wind velocity (m/s). Pasture (p = 0.02) and precipitation (p = 0.01) were significantly associated with the community composition (see Table S3 for corresponding environmental vector correlations).

**
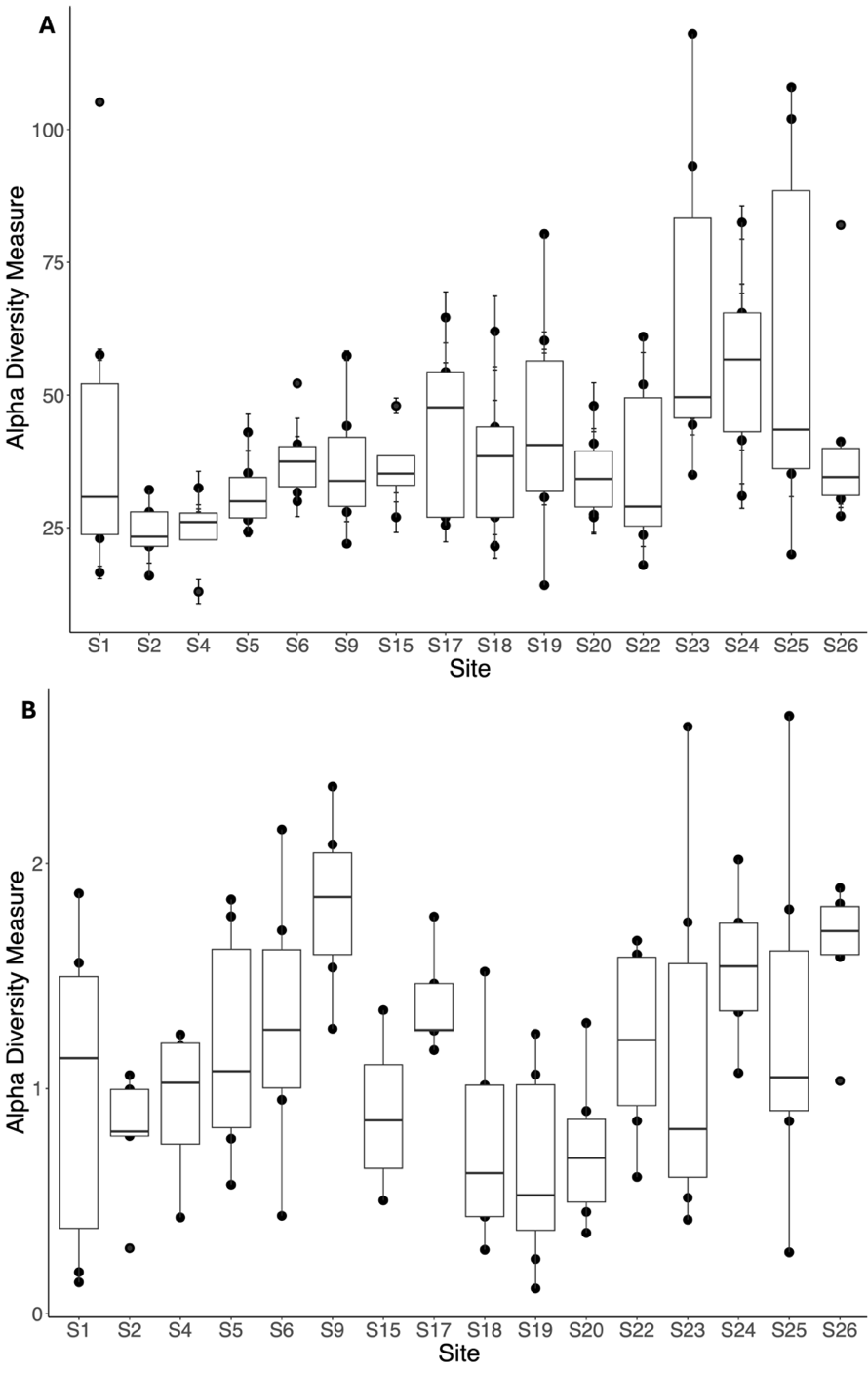
**

**Figure S3.** Alpha diversity measures for *B. terrestris* gut bacteria across 16 sites. (A) Chao1 richness estimates for *B. terrestris* gut bacterial samples per site. All sites were statistically insignificant, *t-test*: p > 0.05 (see Table S3 for all corresponding *t-test* results) (B) Shannon’s diversity indices for *B. terrestris* gut bacterial samples per site. S9 is statistically significant with S19, ANOVA: p = 0.010; S9 is statistically significant with S20, ANOVA: p = 0.032 (Tables S6 and S7). For both plots, boxes represent the median, standard deviation and outliers.

**
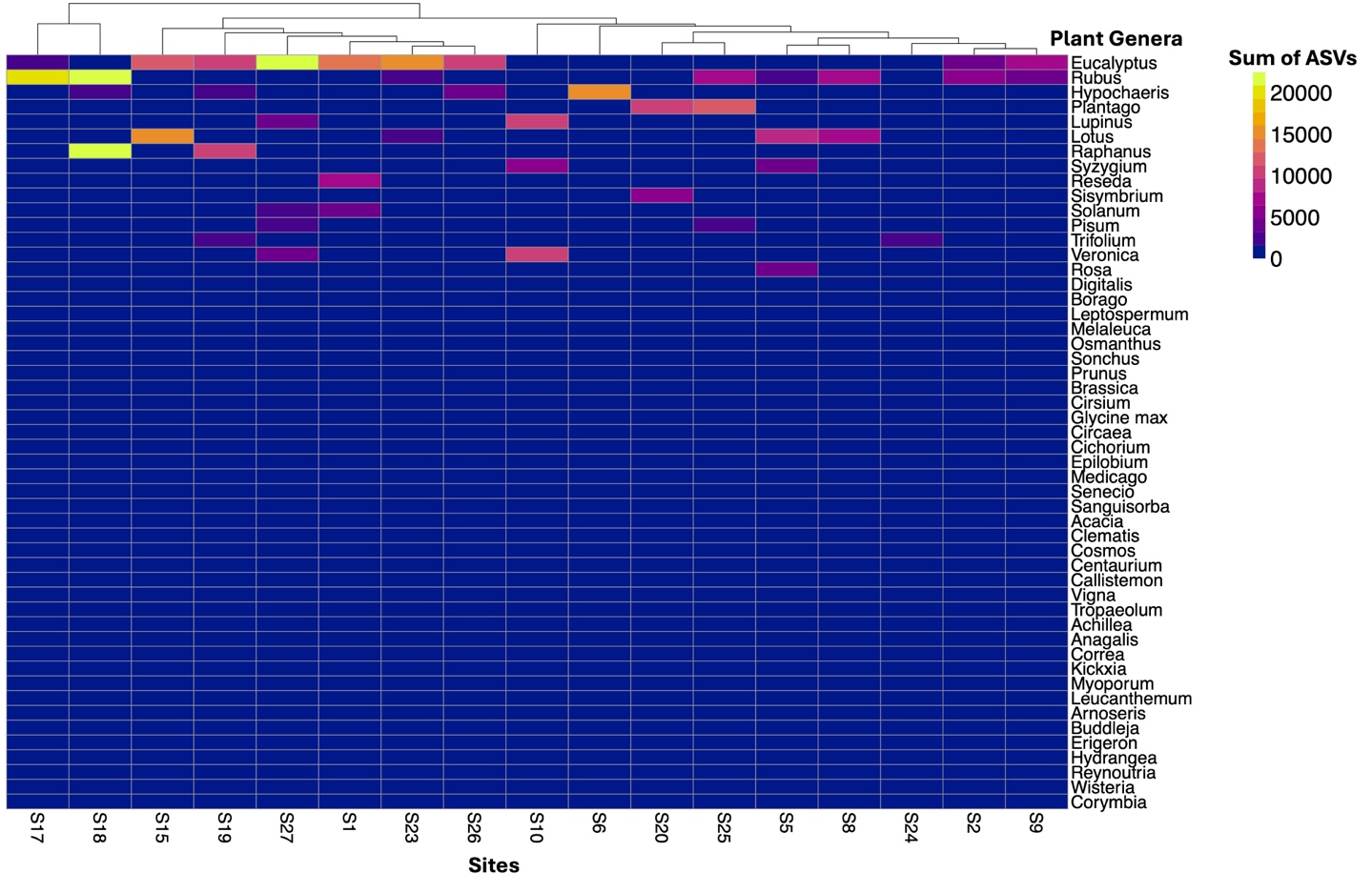
**

**Figure S4.** Heatmap showing all the plant genera identified from the corbicular pollen of *B. terrestris* across 17 sites. The colour scales indicate the sum of amplicon sequence variants (ASVs) under each plant genus. The dendrogram on the top of the heatmap shows the distance or similarities among the sites. The dendrogram is generated using Euclidean distance as the hierarchial clustering measure, which revealed the specific nodes for each site (see Table S2 for categorisation of plant genera)

**Figure S5.** The positive correlation between mean annual precipitation and gut bacterial diversity of *B. terrestris* across Tasmanian sites.

**Figure S6.** (A) Relationship between gut bacterial diversity and pollen diversity of *B. terrestris*. (B) Relationship between *B. terrestris* gut bacterial diversity and average summer wind velocity. Both linear relationships are statistically insignificant (*lm*: p > 0.05). In both plots, the dots represent different Tasmanian sites.

**Figure S7.** Relationship between Shannon’s diversity of corbicular pollen and the six environmental variables which includes: (A) mean annual temperature, (B) mean annual precipitation, (C) percentage of pasture, (D) average summer wind velocity, (E) height of vegetation and (F) percentage of urbanisation. All six linear plots indicate statistically insignificant interactions between pollen diversity and environmental factors (*lm*: p > 0.05). In both plots, the dots represent different Tasmanian sites.

**Figure S8.** The negative correlation between percentage of pasture and the diversity of *B. terrestris* gut bacteria across Tasmanian sites.
